# Localization of fluorescent gold nanoparticles throughout the eye after topical administration

**DOI:** 10.3389/fmed.2025.1557611

**Published:** 2025-03-19

**Authors:** Gabrielle Raîche-Marcoux, Sébastien Méthot, Ange Tchatchouang, Camille Bettoli, Cloé Maranda, Alexis Loiseau, Stéphanie Proulx, Patrick J. Rochette, Emilie Genin, Élodie Boisselier

**Affiliations:** ^1^CHU de Québec Research Center-Université Laval and Department of Ophthalmology and Otolaryngology-Head and Neck Surgery, Faculty of Medicine, Université Laval, Quebec City, QC, Canada; ^2^Université de Bordeaux, CNRS, Bordeaux INP, ISM, UMR 5255, Talence, France

**Keywords:** gold nanoparticles, click chemistry, fluorescence imaging, ophthalmology, biodistribution

## Abstract

The human eye is a highly intricate sensory organ. When a condition requiring treatment occurs, eyedrops, which represent 90% of all ophthalmic treatments, are most frequently used. However, eyedrops are associated with low bioavailability, with less than 0.02% of therapeutic molecules reaching the anterior chamber. Thus, new delivery systems are required to ensure sufficient drug concentration over time at the target site. Gold nanoparticles are a promising avenue for drug delivery; however, they can be difficult to track in biological systems. Fluorescent gold nanoparticles, which have the same ultrastability and biocompatibility as their nonfluorescent counterpart, could act as an effective imaging tool to study their localization throughout the eye after administration. Thus, this study (1) synthesized and characterized fluorescent gold nanoparticles, (2) validated similar properties between nonfluorescent and fluorescent gold nanoparticles, and (3) determined their localization in the eye after topical application on *ex vivo* rabbit eyes. The fluorescent gold nanoparticles were synthesized, characterized, and identified in the cornea, iris, lens, and posterior segment of rabbit eyeballs, demonstrating tremendous potential for future drug delivery research.

## Graphical abstract

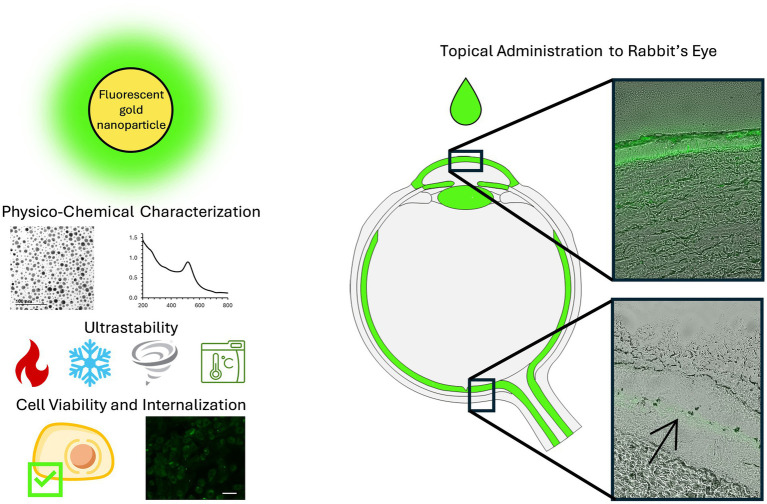

## 1 Introduction

The eye is one of the most complex and sophisticated sensory organs, and the anatomical structure and physiology of the eye are specifically adapted to allow the passage of light (1). Anatomically, the eye is divided into the anterior and posterior segments, which make up one-third and two-thirds of its total dimension, respectively (2).

The anterior segment encompasses the lacrimal apparatus, cornea, conjunctiva, anterior and posterior chambers, iris, ciliary bodies, lens, and aqueous humor (3). In addition, the anterior segment is very exposed to the environment; thus, it is prone to injuries. The corneal tissue features a complex anatomy that provides significant resistance against the entry of foreign substances and microbes, thereby safeguarding vision (4). Blinking, baseline and reflex tearing, and drainage mechanisms help eliminate harmful microbes, foreign particles, and substances, including drug molecules, from the surface of the eye quickly (5–8). The highly dynamic tear film, which has a renewal rate of 1–3 μL/min for an overall volume of 3–6 μL for a typical human eye, adds an additional layer of protection (9). In addition, the unique structure of the corneal tear film, with its lipid, aqueous, and mucoid layers, impedes the rapid absorption of drug molecules by the corneal epithelial cells (10). Thus, considerable efforts have been directed toward enhancing the permeability and retention time of therapeutic substances on the ocular surface (11). Furthermore, creating drug formulations that require less frequent application will greatly improve patient compliance and overall quality of life (12).

The posterior segment, which includes the sclera, choroid, Bruch’s membrane, retina, vitreous humor, and the optic nerve, is vascularized and not easily accessible for noninvasive therapies (13–15). Procedures to control neovascularization in the posterior segment of the eye include laser photocoagulation (16) and photodynamic therapy (17), and, prior to the development of antivascular endothelial growth factor (VEGF) therapy, these methods were the standard treatments to prevent further choroidal neovascularization (18, 19). Despite their success, these treatments have limited effects on overall vision, with reports of choroidal neovascularization recurrences and vision loss (20, 21). In addition, these treatments are not suitable for all patients due to varying individual conditions and disease progressions (22). Intravitreal injections of anti-VEGF agents, e.g., ranibizumab (23), bevacizumab (24), and aflibercept (25), are effective in terms of reversing retinal neovascularization. Intravitreal injections increase local drug concentrations in the vitreous chamber; however, they can be painful and require frequent clinic visits and administration by specialists (26). Complications from repeated intravitreal injections can include retinal detachment, hemorrhaging, retinal toxicity, corneal abrasion, temporary elevation of intraocular pressure, and endophthalmitis (27).

Ocular drug administration is crucial for treating common eye diseases, e.g., glaucoma (28), macular degeneration (29), diabetic retinopathy (30), infections (e.g., conjunctivitis, keratitis, and endophthalmitis) (31), and autoimmune disorders (e.g., Sjögren syndrome and uveitis) (32). Each route of ocular administration (topical, intracameral, intravitreal, or periocular) has its advantages and disadvantages. Indeed, intracameral injections bypass pre-corneal barriers, providing high bioavailability for various classes of active molecules. However, potential complications include the toxic effects of active molecules on corneal endothelial cells and toxic anterior segment syndrome (33). Periocular injections (retrobulbar, peribulbar, sub-Tenon, and subconjunctival) are considered less invasive and associated with fewer potential side effects compared to intravitreal injections. However, scleral barriers to the choroid, combined with elimination through blood and lymphatic flow in the subconjunctival space, lead to lower bioavailability in the retina (34). Eyedrops are the most widely used administration pathway, representing 90% of all ophthalmic treatments (35). Eyedrops are noninvasive and can be self-administered, thereby eliminating further doctor visits, which reduces healthcare system congestion. Eyedrops are applied directly to the desired tissue, and systemic administration of therapeutic agents targeting the eye requires high concentrations, which could lead to toxicity (36). The primary disadvantages of eyedrops are the low bioavailability and patient compliance, which reduces the activity of the active molecule (37). Thus, new ocular delivery systems must be developed to ensure that drugs reach the target site in sufficient concentrations over the prescribed period to achieve the desired therapeutic effect.

Drug delivery systems that utilize nanomaterials can play a pivotal role in targeting ocular tissues for treatment. Using such delivery systems offers several advantages, e.g., promoting drug absorption by enhancing their passage through barrier tissues, controlling their release through local administration, and targeting drug action on specific tissues (38, 39). In recent reviews on nanotechnology-based drug delivery systems in ophthalmology, works on biodistribution of micelles, liposomes, dendrimers, nanosuspensions, nanoemulsions and polymerics nanoparticles are mentioned but studies on gold nanoparticles (AuNPs) are often completely omitted (40–48). Among the reviews citing studies on AuNPs in ophthalmology (34, 49–58), the AuNPs are injected intravenously (59–61), intravitreally (62–65), in the subretinal space (66), administered orally (67), used solely *in vitro* (68–72), used on retinal explants (73), loaded onto contact lenses (74–76) or not used for drug-carrying purposes (77–80).

Mucoadhesion, which is the ability to adhere to mucosal tissues, is a leading strategy for topical administration of nanocarriers on the ocular surface (81), and a recent study demonstrated that AuNPs have mucoadhesive properties; thus, AuNPs are promising candidates for drug delivery systems in ophthalmology (82). In addition to their mucoadhesive properties, AuNPs have unique optical characteristics, e.g., surface plasmon resonance (83) that are beneficial for various biomedical applications (84–89). The polyethylene glycol (PEG) modified AuNPs (AuNPs-PEG_2000_) developed in our laboratory are ultrastable (90) and mucoadhesive; thus, they are highly relevant candidates for drug delivery in ophthalmology (91).

To optimize the potential of these patented AuNPs-PEG_2000_ (CA3043775) as drug delivery systems, experiments were conducted to better understand their drug loading and drug release properties (92). The next step was an investigation into their behavior and distribution in the eye after topical application. However, they are currently difficult to track with currently available technologies without damaging the biological tissues; thus, we sought to modify them to emit fluorescence without impacting their other properties. This would allow the new fluorescent AuNPs-PEG_2000_-Nap to act as an imaging tool in place of the nonfluorescent AuNPs-PEG_2000_. Therefore, the goal of this study was to (1) synthesize and characterize the fluorescent AuNPs-PEG_2000_-Nap, (2) validate similar properties between the nonfluorescent and fluorescent AuNPs-PEG_2000_ and AuNPs-PEG_2000_-Nap, and (3) determine their localization in the eye after topical application using *ex vivo* rabbit eyes. The results of this study are expected to facilitate better understanding of the behavior of the AuNPs-PEG_2000_ in physiological conditions and their potential as drug delivery systems in ophthalmology.

## 2 Materials and methods

### 2.1 Materials

Gold chloride trihydrate (HAuCl_4_∙3H_2_O), sodium borohydride (NaBH_4_), chlorohydric acid (HCl), nitric acid (HNO_3_), sodium chloride (NaCl), potassium chloride (KCl), sodium phosphate dibasic (Na_2_HPO_4_), potassium phosphate monobasic (KH_2_PO_4_), basic fuchsine (pararosaniline hydrochloride), sodium metabisulfite, isopropanol, and acetonitrile were all purchased from VWR International (Ville Mont-Royal, QC, Canada). Anhydrous copper(II) sulfate (CuSO_4_), ascorbic acid, 4-bromo-1,8-naphthalimide anhydride, 2-methoxyethylamine, sodium azide, absolute ethanol, petroleum ether, and ethyl acetate were purchased from Sigma-Aldrich (St. Louis, MO, United States). Polyethylene glycol methyl ether thiol with a molecular weight of 2000 g/ mol (referred to as HS-PEG_2000_) was purchased from Laysan Bio (Arab, AL, United States). Polyethylene glycol alkyne thiol (referred to as HS-PEG_2000_-ALK) was purchased from Biopharma PEG (Watertown, MA, United States). All glassware used to synthesize the AuNPs was washed thoroughly with aqua regia (3:1 HCl:HNO_3_) and rinsed with nanopure water prior to experimentation. Mucins from the bovine submaxillary gland were purchased from Cedarlane Laboratories (Burlington, ON, Canada). 3-(4,5-dimethylthiazol-2-yl)-5-(3-carboxymethoxyphenyl)-2-(4-sulfophenyl)-2H-tetrazolium (MTS) was obtained from Promega (Madison, WI, United States). Research-grade human eyes were provided by the “Ocular tissue for vision research” infrastructure of the Vision Sciences Research Network, in collaboration with our local eye bank (Banque d’Yeux du Center Universitaire d’Ophtalmologie, Québec, QC, Canada) and Héma-Québec (Québec, QC, Canada) with next of kin consent. This study followed the Declaration of Helsinki and was approved by the CHU de Québec-Université Laval ethics committee (DR-002-955). Eight healthy corneas (age 55–66 years; median 61.5; SD ± 4.6) were used for the characterization of the fluorescence emission, and three (aged 44, 52, and 71 years) were used for hCEC cell isolation and culture, as reported previously (93, 94). Irradiated human fibroblasts (iHFL) were isolated from the foreskin of a 10-day-old donor (95, 96). The OCT compound was obtained from Sakura Finetek (Torrance, CA, United States). Albino rabbit heads (*N* = 6) were obtained from the Rolland Pouliot & Fils slaughterhouse (Saint-Henri-de-Lévis, QC, Canada).

### 2.2 Synthesis and characterization of 4-azido-N-(2-methoxyethyl)-1,8-naphthalimide

Here, 2-methoxyethylamine (2.59 mL, 29.9 mmol, 2.07 eq) was added to a solution of 4-bromo-1,8-naphthalimide anhydride (4 g, 14.4 mmol, 1 eq) in 50 mL of absolute ethanol. The mixture was refluxed overnight under stirring and then cooled to 0°C by immersing the flask in an ice bath. The solid was collected by filtration to obtain the key intermediate 4-bromo-N-(2-methoxyethyl)-1,8-naphthalimide (4,67 g, 97% yield).

^1^H NMR (300 MHz, CDCl_3_) *δ*: 8.66 (d, J = 7.3 Hz, 1H, ArH), 8.55 (d, J = 9.0 Hz, 1H, ArH), 8.41 (d, J = 7.8 Hz, 1H, ArH), 8.03 (d, J = 7.8 Hz, 1H, ArH), 7.84 (t, J = 7.6 Hz, 1H, ArH), 4.43 (t, J = 5.8 Hz, 2H, CH_2_), 3.73 (t, J = 5.8 Hz, 2H, CH_2_), 3.37 (s, 3H, CH_3_).

Then, NaN_3_ (775 mg, 11.9 mmol, 1.5 eq) was slowly added to a solution of the key intermediate (3 g, 7.95 mmol, 1 eq) in 114 mL of dimethylformamide. The mixture was heated at 70°C under stirring for 2 h. The solvent was removed under reduced pressure. The residue was purified by chromatography on silica gel (petroleum ether/ethyl acetate: gradient from 8:2 to 5:5). 4-azido-N-(2-methoxyethyl)-1,8-naphthalimide was obtained as a pale yellow solid (2.11 g, 84% yield).

^1^H NMR (300 MHz, CDCl_3_) *δ*: 8.65 (d, J = 7.3 Hz, 1H, ArH), 8.59 (d, J = 7.9 Hz, 1H, ArH), 8.44 (d, J = 8.5 Hz, 1H, ArH), 7.74 (t, J = 7.9 Hz, 1H, ArH), 7.47 (d, J = 7.9 Hz, 1H, ArH), 4.44 (t, J = 5.9 Hz, 2H, CH_2_), 3.73 (t, J = 5.9 Hz, 2H, CH_2_), 3.38 (s, 3H, CH_3_).

The results of the NMR analysis are in agreement with the published values for 4-bromo-N-(2-methoxyethyl)-1,8-naphthalimide and 4-azido-N-(2-methoxyethyl)-1,8-naphthalimide (97). They were recorded using a spectrometer Avance 300 MHz (Bruker). The obtained ^1^H NMR spectra are provided as Supplementary Material (Supplementary Figures 1, 2).

### 2.3 Synthesis of HS-PEG_2000_-nap

In a 25-mL Erlenmeyer flask, 0.02 g of HS-PEG_2000_-ALK (0.005 mmol) was dissolved in 15 mL of acetonitrile. The 4-azido-N-(2-methoxyethyl)-1,8-naphthalimide, 0.0148 g (0.050 mmol) was added to the mixture. Copper sulfate (0.00160 g; 0.005 mmol) and ascorbic acid (0.00176 g; 0.005 mmol) were then added to the Erlenmeyer flask. The mixture was kept at 22°C and shaken using an orbital shaker at 250 rpm overnight in darkness.

### 2.4 Synthesis of AuNPs-PEG_2000_-nap 1%

Stirring at 400 rpm, 250 μL of an aqueous solution of 0.1 g/mL of gold chloride trihydrate (0.06 mmol) was added to 15 mL of a 1:1 acetonitrile:isopropanol mix. 0.0099 g of a HS-PEG_2000_ and 750 μL of HS-PEG_2000_-Nap solution obtained previously were added to 30 mL of isopropanol prior to addition to the gold solution. The resulting solution was mixed for 1 h at 400 rpm. While increasing the mixing speed to 700 rpm, a fresh solution of 0.028 g of NaBH4 (0.74 mmol) in 10 mL of ice-cold water was added dropwise to the gold solution using a peristaltic pump (1 mL/min). The mixture was then stirred at 400 rpm for 3 h to allow the nanoparticles to grow and stabilize in the dark. The solution was evaporated using a rotary evaporator under reduced pressure. Then, 10 mL of water was added to resuspend the nanoparticles. The purification of the nanoparticles performed by dialysis (molecular weight cutoff: 16,000 g/mol) for 7 days, changing the dialysate at least four times per day. After 7 days, the AuNPs-PEG_2000_-Nap 1% were transferred to a polycarbonate centrifuge tube (#355631) and centrifuged with a 70 Ti rotor using the Optima L-90 K Ultracentrifuge from Beckman Coulter Inc. (Indianapolis, IN, United States) at 220,000 g for 18 h. Then, the supernatant was removed and replaced with ultrapure water. The AuNPs-PEG_2000_-Nap 1% were resuspended and moved back to a new dialysis bag with the same molecular weight cutoff for seven supplementary days (dialysates changed four times per day). The AuNPs-PEG_2000_-Nap 1% were then centrifugated again, and the supernatant was changed with ultrapure water. To determine the concentration of the AuNPs in the resultant solution, a known volume of the solution was freeze-dried and weighed (*n* = 3).

### 2.5 Synthesis of AuNPs-PEG_2000_-nap 100%

To obtain AuNPs-PEG_2000_-Nap 100%, 250 μL of an aqueous solution of 0.1 g/mL of gold chloride trihydrate (0.06 mmol) was added to 7.5 mL of the previously synthesized HS-PEG_2000_-Nap and 7.5 mL of isopropanol. All the following steps remain the same as those for the AuNPs-PEG_2000_-Nap 1%.

### 2.6 Characterization of AuNPs-PEG_2000_-nap

#### 2.6.1 UV–visible spectroscopy

Here, a quartz cuvette (pathlength: 1 mm × 10 mm) from Hellma (#104.002-QS) was used (Markham, ON, Canada). The UV–visible spectra were collected from 200 to 800 nm using a Cary Eclipse 50 Bio UV–vis spectrophotometer from Varian (Winnipeg, MB, Canada).

#### 2.6.2 First derivative of plasmon band spectra

The UV–visible spectra were normalized using the intensity of the peak of the plasmon band of AuNPs-PEG_2000_-Nap 1% (*λ* = 517.5 nm) and AuNPs-PEG_2000_-Nap 100% (λ = 519.0 nm). Then, the data between 430 nm and 630 nm were derived for AuNPs-PEG_2000_-Nap 1% and AuNPs-PEG_2000_-Nap 100%. Here, the inflection point of the derivative was used to determine the position of the plasmon band peak (98–100).

#### 2.6.3 Fluorescence spectroscopy

The fluorescence spectra of the AuNPs-PEG_2000_-Nap 1 and 100% samples were obtained using a Fluorolog-3 instrument (Horiba). A 5 × 5 mm light path quartz cuvette from Hellma Analytics was used (#111.057-QS). The excitation wavelength exhibited optimal results at 420 nm; thus, the scans for emission were recorded from 490 to 600 nm.

#### 2.6.4 Transmission electron microscopy

Copper grids covered with a vaporized carbon film were purchased from Ted Pella (California, United States). AuNPs-PEG_2000_-Nap 1 and 100% were diluted to a concentration of approximately 0.01 mg/mL, and a 5-μL droplet was deposited on each grid and left to dry overnight. Image acquisition was performed using a JEM 1230 from JEOL Ltd. (Tokyo, Japan). Here, the voltage was set to 80 kV, and a 50,000× zoom factor was used. To characterize the size of the gold core, at least 3,000 AuNPs-PEG_2000_-Nap 1% and AuNPs-PEG_2000_-Nap 100% were counted and analyzed for each sample using the ImageJ software.

#### 2.6.5 Dynamic light scattering

DLS measurements were performed using the NanoBrook Omni particle analyzer from Brookhaven Instruments Corporation (Holtsville, NY, United States). Each AuNPs-PEG_2000_-Nap was diluted to a concentration of 0.033 mg/ mL in PBS (1X). Prior to analysis, the samples were filtered through a 0.2-μm pore size filter. The apparatus was set to an angle of 173° at 25°C. After an equilibrium time of 2 min, 10 measurements of 120 s were performed for each sample. BI-SCP disposable plastic cuvettes from Brookhaven Instruments Corporation (Holtsville, NY, United States) were used for the analysis. The treatment of the size distribution was performed using the CONTIN algorithm.

#### 2.6.6 Elemental analysis

Elemental analysis was performed using an ICP-OES, which requires the oxidation of gold prior to analysis. Here, AuNPs-PEG_2000_-Nap 1 and 100% were diluted to a 1:9.5 aqua regia to water ratio and a final AuNP concentration of 0.20 mg/mL. Blank standards containing 5% aqua regia were also analyzed to confirm the absence of contamination specific to the method. The sample preparation was performed in 15-mL tubes from Sarstedt (#62.554.100) (Montreal, QC, Canada). The tubes were heated to 90°C, with care being taken to avoid boiling. The tube caps were left on the tubes and closed by a quarter of a turn. The apparatus used in the experiments was the ICP-OES-5110 (Agilent) in radial mode (Mississauga, ON, Canada). The calibration methods of the yttrium internal standard and standard addition were performed to prevent matrix effects. Acquisition was achieved at 242.8 nm and 181.9 nm for gold and sulfur quantification, respectively.

#### 2.6.7 Molecular weight approximation

The molecular weight (MW) of the AuNPs was determined approximatively following a previously published method (101). Briefly, while assuming monodisperse spherical AuNPs, the volume (V) of the gold core can be estimated as follows.


$$V=\frac{4\pi \times {\left(\mathrm{radius in}\;\overset{{}^{\circ}}{A}\right)}^{3}}{3}$$

The number of gold atoms found in the metallic core, n(Au), can be estimated as follows.


$$n\left(Au\right)=\frac{V\;\mathrm{in}\;{\overset{{}^{\circ}}{A}}^{3}}{\;17\;{\overset{{}^{\circ}}{A}}^{3}}$$

With the MW of the Au and S, the number of thiol groups n(S) on the surface can be estimated as follows.

$$\frac{n\left(Au\right)\times MW\left(Au\right)}{\%Au\;\mathrm{atoms}}=\frac{n\left(S\right)\times MW\left(S\right)}{\%S\;\mathrm{atoms}}$$

Finally, the MW of AuNPs-Nap 1 and 100% can be estimated using the following equation.


$$\begin{array}{l} MW\left(\mathrm{AuNPs}-{\mathrm{PEG}}_{2000}-\mathrm{Nap}\;1\%\right)=\left[n\left(Au\right)\times MW\left(Au\right)\right]\\ +0.01\left[n\left(S\right)\times \left(MW\left(\mathrm{PEG}-\mathrm{Nap}\right)\right)\right]+0.99\left[n\left(S\right)\times MW\left(\mathrm{PEG}\right)\right] \end{array}$$

$$\begin{array}{l} MW\left(\mathrm{AuNPs}-{\mathrm{PEG}}_{2000}-\mathrm{Nap}\;100\%\right)\\ =\left[n\left(Au\right)\times MW\left(Au\right)\right]+0.01\left[n\left(S\right)\times \left(MW\left(\mathrm{PEG}-\mathrm{Nap}\right)\right)\right] \end{array}$$

#### 2.6.8 Graft density approximation

The graft density $\rho_{g}$ was determined using the calculated number of ligands n(S) used in the approximation of the MW. The surface of the metallic core A, assuming monodisperse spherical nanoparticles, can be calculated as follows.


$$A=4\pi \times {\left(\mathrm{radius in}\;\mathrm{nm}\right)}^{2}$$

Thus, the graft density can be evaluated as follows.


$$\rho_{g}=\frac{n\left(S\right)}{A}$$

### 2.7 Biolocalization in human corneas

The human corneas were rinsed with PBS to remove the storing liquid and placed on a small support (epithelium side up). A punched out 6-mm diameter rubber stencil was centered on the cornea to ensure that the deposited drop remained on the cornea and did not flow over and to the side after application. A drop (50 μL, 1 mg/mL) of AuNPs-PEG_2000_, AuNPs-PEG_2000_-Nap 1%, and AuNPs-PEG_2000_-Nap 100% were placed on top of the corneas. Then, the corneas were placed in the dark for 2 h before being prepared for tissue imaging.

### 2.8 Ultrastability assays

The protocol used to qualify the ultrastability properties strictly followed the previously established protocol (90).

### 2.9 Quantification of mucoadhesion

The protocol used to quantify the adsorbed mucins on the surface of the AuNPs strictly followed a previously published PAS coloration protocol (101).

### 2.10 MTS assays

MTS viability assay was performed according to the manufacturer’s instructions (Promega, Madison, WI). Briefly, hCECs (1 × 10^4^) in corneal epithelium medium [Dulbecco–Vogt modification of Eagle’s medium (Gibco, Waltham, MA, United States) with Ham’s F12 (3:1) (Life Technologies, Carlsbad, CA, United States), supplemented with 5% FetalClone II serum (HyClone, Logan, UT, United States), 5 μg/mL insulin (SAFC Bioscience, Lenexa, KS, United States), 0.4 μg/mL hydrocortisone (Teva, Toronto, ON, Canada), 10 ng/ mL epidermal growth factor (R&D Systems, Oakville, ON, Canada), 10^−10^ mol/ L cholera toxin (Sigma-Aldrich, St. Louis, MO, USA), 100 μg/mL Penicillin (Fresenius Kabi, Homburg, Germany), and 25 μg/mL Gentamycin (Galenova, Saint-Hyacinthe, QC, Canada)] and incubated at 37°C for 4 h prior to the addition of AuNPs (AuNPs-PEG_2000_ and AuNPs-PEG_2000_-Nap 1%, at concentrations of 0, 0.001, 0.1, 0.25, 0.50, 0.75 and 1.00 μM). After 18 h, MTS (2 mg/mL) was added to each well, and the plates were incubated at 37°C for 1 h. Then, the optical density (OD) was measured using a microplate reader (Biorad Model 550, Mississauga, ON, Canada) at a wavelength of 490 nm. OD of the blank solution (AuNPs, no cells) was subtracted, and results presented relative to the control (0 μM AuNPs, which represents 100% viability). Experiments were performed using 3 different cell populations, in triplicate.

### 2.11 Internalization assays

hCECs (0.3 × 10^6^ cells) were plated directly in six-well plates (9.6 cm^2^) in the corneal epithelium medium described above. Before the cells reached confluence, AuNPs-PEG_2000_ and AuNPs-PEG_2000_-Nap 1% (500 μL, 1 mg/mL, diluted with PBS) were added. After a waiting time of 30 min, the cells were rinsed with PBS before image acquisition was performed using an Axio Imager Z2 Upright Microscope (Carl Zeiss, North York, ON, Canada).

### 2.12 Biolocalization with rabbit eyes

The rabbit eyeballs were removed from the skull and placed on a small support (cornea side up). A 20-mm diameter rubber gasket well was centered on the cornea to ensure that the deposited drop remained on the cornea and did not flow onto the globe after application. A drop (50 μL, 1 mg/mL) of AuNPs-PEG_2000_-Nap 1% was placed in the middle of the stencil. Here, two waiting times were tested, i.e., 2 h [to maximize the interactions mucins-AuNPs, as previously demonstrated (101)] and 3 min (*n* = 3 eyes per condition). For the 2-h waiting time, the eyeballs were placed in the dark before the drop was applied. After 2 h, the globes were prepared for imaging. For the 3-min waiting time, which mimics the renewal rate of the tear film (9), the eyeballs were washed thoroughly with PBS to ensure that no AuNPs-PEG_2000_-Nap 1% remained on the ocular surface. The eyeballs were then placed in the dark for 2 h for comparison with the first waiting time before tissue preparation for imaging. The controls (*n* = 3 eyes) received a drop of PBS and were placed in the dark for 2 h prior to tissue preparation.

### 2.13 Tissue preparation for imaging

The dissections were performed to separate the corneas, irises, lenses, and posterior segment of the eyes, and to avoid all cross-contamination. The centers of all corneas were cut using a 6-mm biopsy punch before being placed (endothelium down) in an OCT compound from Sakura Finetek (Torrance, CA, United States). The cut samples were then stored at 80°C at least overnight before making cuts using a cryostat microtome (Leica, Concord, Canada). The irises were placed flat with the posterior epithelium down in the OCT compound, and the lens was placed with the epithelium side up. A portion of the posterior segment was removed using a 10-mm biopsy punch to facilitate manipulation. The punched sample was put in the OCT compound. All samples were stored at 80°C at least overnight before making microtome cryostat cuts. Prior to being placed on microscope slides, the samples of the corneas (human and rabbit), iris, and posterior segments were sliced (thickness: 8 μm), and the lens samples were prepared with a thickness of 16 μm. Upon microscopic examination, samples without AuNPs were used to determine the exposure time that prevented the background signal and endogenous fluorescence in the sample.

## 3 Results and discussion

The development of effective delivery systems relies heavily on optimizing formulation and process parameters and thoroughly characterizing the physicochemical and biological properties of nanocarriers. In ocular drug delivery, key factors to assess include particle size and distribution, surface charges, encapsulation efficiency, drug loading capacity, drug release profiles, uptake mechanisms, stability, and safety or toxicity (102). Additionally, the delivery system must meet specific ocular requirements such as sterility, osmolality, pH, surface tension, and viscosity to ensure compatibility and efficacy. Important properties, like particle size and polydispersity index, are critical for the physical stability of nanocarriers. For ocular formulations, particles larger than 10 μm are generally unsuitable (103). Smaller, monodisperse particles are preferred for their enhanced stability, biodistribution (104), and reduced risks of instability issues like sedimentation during storage (105). Their small size facilitates faster penetration into the tear film’s mucin layer, reduces irritation, and enhances uptake by corneal epithelial cells (106). While smaller nanoparticles show greater absorption into the aqueous humor, they are cleared more quickly from tear fluid (107). The shape and surface morphology of nanoparticles also influence their biodistribution, cellular uptake, and toxicity. Spherical nanoparticles are generally more effective in enhancing drug performance compared to other shapes like cubes or rods (108). Surface modifications are also important factors for biodistribution: different polymeric nanoparticles, and even different polymeric shells will have different biodistribution profiles in the anterior and posterior segment (109, 110).

To investigate the localization of nonfluorescent AuNPs after eyedrop application, a strategy was required to determine their trajectory accurately. As previously synthesized, the gold core combined into the PEGylated ligands created a furtive drug delivery system (111, 112); thus, the goal was to minimally modify the AuNPs-PEG2000, that are small, spherical, stable and biocompatible, to generate an imaging tool that would be visible in microscopy and replicate the nonfluorescent AuNPs-PEG2000 behavior. Here, fluorescence microscopy was selected for its sensitivity (113), its compatibility with animal and human tissues (114), and the minimal modification required to the AuNPs-PEG2000 to make them fluorescent (115, 116). Once the characteristics of the AuNPs-PEG2000 and fluorescent AuNPs-PEG2000-Nap were determined to be equivalent, internalization assays were conducted prior to performing biolocalization experiments.

### 3.1 Synthesis and physicochemical characterization of fluorescent AuNPs

#### 3.1.1 Synthesis of fluorescent AuNPs-PEG_2000_-nap

**SCHEME 1:**
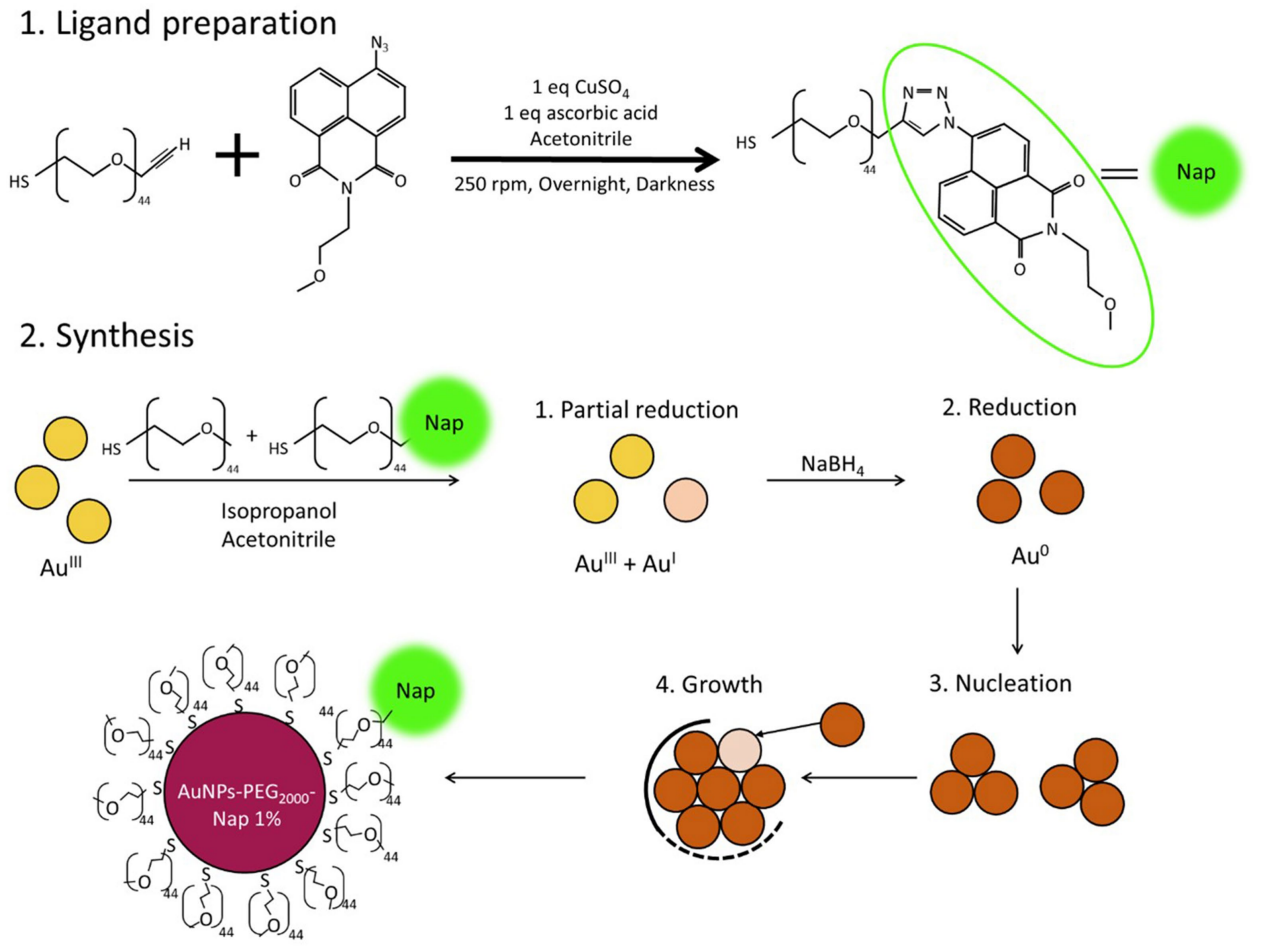
AuNPs-Nap synthesis. The proportion between the HS-PEG_2000_-Nap and HS-PEG_2000_ varies from 1 to 100%. For the experimental details, refer to the description in the Materials and Methods.

To obtain AuNPs-PEG_2000_-Nap, the first step involved synthesizing the fluorescent ligand (HS-PEG_2000_-Nap; Scheme 1). Here, a heterobifunctional PEGylated ligand (HS-PEG_2000_-ALK), with an alkyne functional group on one end (dedicated to the click reaction) and a thiol group (-SH) on the other, was employed to covalently bond the naphthalimide (Nap) fluorophore and coordinate with the Au atoms of the core (through the thiol group). The click reaction (117) was performed with stoichiometric equivalents of reagents, and the azido-naphthalimide equivalent was 10 times the molar equivalent of the ligand. The advantage of this molecule lies in its difference in fluorescence emission before and after the click reaction (118). In fact, prior to the click reaction, the azido-naphthalimide was very weakly fluorescent, whereas it became a strong fluorophore after the formation of the triazole heterocycle.

Here, the synthesis process was a modified version of the Brust–Schiffrin synthesis (119) based on specific experimental conditions leading to small ultrastable AuNPs (90). Two types of AuNPs-PEG_2000_-Nap were obtained with this synthesis, i.e., AuNPs-PEG_2000_-Nap 1% and AuNPs-PEG_2000_-Nap 100%, where 1 and 100% of the ligands used for the synthesis were the HS-PEG_2000_-Nap, respectively. For the AuNPs-PEG_2000_-Nap 1%, the remaining 99% of the ligands were monofunctional HS-PEG_2000_ (90). Once the fluorescent HS-PEG_2000_-Nap was obtained, it was added in different proportions in the synthesis of the AuNPs. Mixing the PEGylated ligands with chloroauric aid (HAuCl_4_) caused a partial reduction of the Au atoms. Note that the success of this synthesis is dependent on the balance between the crystal growth and the ligand capping speeds, i.e., if the crystal growth outpaces the ligand capping speed, the particles will not stabilize properly and will precipitate. In addition, acetonitrile in the reaction mixture slows the crystal growth because its nitrogen atoms partially stabilize the Au core growth. Furthermore, NaBH_4_ exhibits lower reducing strength in acetonitrile compared to water, which further slows the crystal growth. This slower growth allows for better capping and results in more stable AuNPs (120).

Prior to characterization, the synthesis was purified via dialysis and precipitation by ultracentrifugation to further change the dialysate (refer to the Materials and Methods).

#### 3.1.2 Physicochemical characterization of AuNPs-PEG_2000_-nap 1 and 100%

The UV–visible spectra showed that the surface plasmon resonance absorbance peaks for AuNPs-PEG_2000_-Nap 1% and AuNPs-PEG_2000_-Nap 100% are centered at 517.5 nm and 519.0 nm, respectively (Table 1). In addition, the elemental analysis via ICP-OES revealed varying weight percentages of gold and sulfur in the AuNPs. Here, AuNPs-PEG_2000_-Nap 1% contained 48.5% gold and 0.41% sulfur, and AuNPs-PEG_2000_-Nap 100% contained 62.5% gold and 0.67% sulfur (Table 1). The TEM observations showed that the gold core diameters were 6.0 ± 3.0 nm and 4.8 ± 2.4 nm for the AuNPs-PEG_2000_-Nap 1% and AuNPs-PEG_2000_-Nap 100%, respectively (Table 1). Based on the metallic core diameters and elemental compositions, the molecular weights were calculated to be 2,002, 334 g/mol for AuNPs-PEG_2000_-Nap 1% and 1,185,970 g/mol for AuNPs-PEG_2000_-Nap 100% (Table 1). DLS measurements showed hydrodynamic diameters of 29 ± 1 nm for AuNPs-PEG_2000_-Nap 1% and 34.0 ± 0.6 nm for AuNPs-PEG_2000_-Nap 100% (Table 1). In addition, the graft density for AuNPs-PEG_2000_-Nap 1% was 3.05 ligands per nm^2^ of gold core, and that for the AuNPs-PEG_2000_-Nap 100% was 3.10 ligands per nm^2^ of gold core (Table 1).

**Table 1:** Characterization of AuNPs-PEG_2000_-Nap 1% and AuNPs-PEG_2000_-Nap 100%.

	AuNPs-PEG_2000_-Nap 1%	AuNPs-PEG_2000_-Nap 100%
Position of plasmon band peak (nm)^a^	517.5	519.0
% Au atoms^b^	48.5	62.5
% S atoms^b^	0.41	0.67
Core diameter (nm)^c^	6.0 ± 3.0	4.8 ± 2.4
Hydrodynamic diameter (nm)^d^	29 ± 1	34.0 ± 0.6
Number of ligands^e^	345	224
Molecular weight (g/mol)^f^	2,002,334	1,185,970
Graft density (number of ligand/nm^2^)^f^	3.05	3.10

a The position of the plasmon band peak was obtained using ultraviolet (UV)-visible spectroscopy.

b % Au and S atoms were determined by elemental analysis using inductively coupled plasma optical emission spectroscopy (ICP-OES).

c The core diameters were determined by transmission electron microscopy (TEM).

d The hydrodynamic diameters were determined by dynamic light scattering (DLS).

e The number of ligands was calculated per AuNPs-PEG_2000_-Nap.

f The molecular weights and graft density were calculated theoretically. Experimental and calculation details are described in the Materials and Methods Section.

The core diameter and number of ligands were the highest for AuNPs-PEG_2000_-Nap 1%, and the percentage of sulfur atoms was the lowest, which can be attributed to the varying behavior of the AuNPs based on their molecular weights, especially during the stabilization of the gold core. In water, PEG alone can adopt Gaussian coil or flat plate configurations (121), and it stabilizes molecules or proteins with wrapped or expanded configurations (122). During AuNPs-PEG_2000_-Nap growth (refer to Scheme 1), the utilization of these two types of PEG (HS-PEG_2000_-Nap and HS-PEG_2000_) in different proportions can lead to different behaviors, resulting in the core diameters ranging from 6. 0 ± 3.0 nm to 4.8 ± 2.4 nm, which are still quite similar on the nanometer scale. It is likely that HS-PEG_2000_-Nap experiences more steric hindrance, thereby reducing the thiol group’s ability to reach the gold core. This steric hindrance may also enhance stabilization, producing smaller cores than expected. Consequently, the smaller gold core of AuNPs-PEG_2000_-Nap 100% leads to a smaller number of stabilizing ligands around the nanoparticles, thereby making AuNPs-PEG_2000_-Nap 1% have the largest gold core diameter and number of ligands. With the largest core diameter, AuNPs-PEG_2000_-Nap 1% contained 6,652 gold atoms, which is significantly more than the AuNPs-PEG_2000_-Nap 100%, which had 3,406 gold atoms. However, for AuNPs-PEG_2000_-Nap 1%, this only represents 0.41% by mass despite having 345 sulfur atoms. The graft density showed very similar numbers of ligands per nm^2^ of gold core surface for both AuNPs-PEG_2000_-Nap 1 and 100%, i.e., 3.05 and 3.10 ligands/nm^2^, respectively.

**Figure 1:**
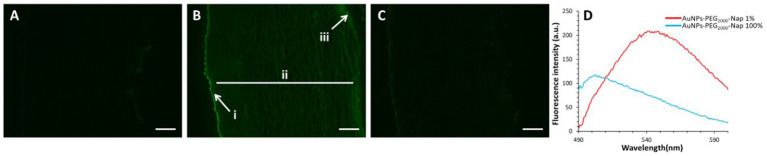
Characterization of the fluorescence emission of AuNPs-PEG2000 and AuNPs-PEG2000-Nap using *ex vivo* human corneas and fluorescence spectroscopy. **(A)** AuNPs-PEG2000 were deposited on a research-grade human cornea to be used as a control. **(B)** AuNPs-PEG2000-Nap 1% were placed on a human cornea, where i is the Bowman’s layer, ii is the stroma, and iii is the Descemet’s membrane. **(C)** AuNPs-PEG2000-Nap 100% were placed on a human cornea. **(D)** The fluorescence emission spectra of AuNPs-PEG2000-Nap 1 and 100%. Scale bar: 5 μm.

Continuing the physical characterization of AuNPs-PEG_2000_-Nap 1 and 100%, the fluorescence emission of the nanoparticles was tested with *ex vivo* human corneas (Figure 1) to identify the best AuNPs-PEG_2000_-Nap for colocalization experiments. A drop (50 μL, 1 mg/mL) of AuNPs-PEG_2000_ (Figure 1A), AuNPs-PEG_2000_-Nap 1% (Figure 1B), and AuNPs-PEG_2000_-Nap 100% (Figure 1C) was deposited on the top of the human corneas and incubated at room temperature for 2 h before being prepared for image acquisition (Materials and Methods). The fluorescence emission spectra of AuNPs-PEG_2000_-Nap 1% and AuNPs-PEG_2000_-Nap 100% (Figure 1D) were also obtained in water. The AuNPs-PEG_2000_-Nap 1% (λ_max, emission_ = 544 nm) had the highest fluorescence emission intensity and was the most visible in the cornea. In fact, AuNPs-PEG_2000_-Nap 1% could be found in the Bowman’s layer, throughout the entire stroma and the Descemet’s membrane, with a stronger affinity with the Bowman’s layer. For AuNPs-PEG_2000_-Nap 100% (λ_max, emission_ = 503 nm), the fluorescence was less discernible in the cornea than with AuNPs-PEG_2000_-Nap 1%. The difference in the fluorescence emission spectra could hypothetically come from the difference in the steric hindrance between AuNPs-PEG_2000_-Nap 1% and AuNPs-PEG_2000_-Nap 100% (123). The presence of Nap groups on all 224 PEGylated ligands of AuNPs-PEG_2000_-Nap 100% could cause steric hindrance, thereby not permitting the Nap group free rotations and reducing the extent of the intramolecular charge transfer, which could explain the blue shift observed in the fluorescence spectra (Figure 1D) (124, 125). The difference in the emission intensities is likely due to aggregation-caused quenching. For the AuNPs-PEG_2000_-Nap 100%, the aromatic Nap fluorophores are closer and can give rise to intermolecular interactions, e.g., *π*-π stacking, which commonly results in reduced fluorescence (126). Between the two fluorescent AuNPs-PEG_2000_-Nap 1 and 100%, the AuNPs-PEG_2000_-Nap 1% appear to present better fluorescent properties for further physiological experiments. The AuNPs-PEG_2000_-Nap could potentially benefit from fine-tuning experiments to investigate the number of Nap groups required to realize optimal fluorescence while modifying the other AuNP properties minimally.

### 3.2 Biological properties of AuNPs-PEG2000-nap

With the goal of using AuNPs-PEG2000 as ocular drug delivery systems, these nanoobjects must exhibit several critical biological properties, e.g., ultrastability under conditions mimicking formulation steps and the physiological environment, mucoadhesion, and biocompatibility. The following three sections describe the biological properties of the AuNPs and their behavior in the presence of human corneal epithelial cells (hCEC).

#### 3.2.1 Ultrastability

Frequently, the weak colloidal stability of AuNPs excludes their utilization in specific fields (127). For example, instability in physiological buffering salts restricts the utilization of many types of nanoparticles for biomedical applications [e.g., drug delivery (128), gene therapy (129), biosensing (130), and imaging (131)] because blood is a rich and highly ionic media (132). In addition, for nanoparticles, the loss of colloidal stability can result in precipitation, reshaping, corrosion, and, most importantly, the loss of key physicochemical properties like delivery, optoelectronic, biocompatibility, biodegradability, magnetic, or catalytic abilities (133). Thus, it is crucial to preserve the original size, shape, structure, composition, and aggregation state of the nanoparticles in biological environments for the required duration to achieve the intended use (134). This preservation is essential for maintaining the functionality of nanoparticles and preventing undesired effects (135). Given that nonfluorescent AuNPs-PEG_2000_ can withstand harsh conditions (90), e.g., several cycles of freeze-drying, heating, ultracentrifugation, and autoclave sterilization, the goal was to verify that the fluorescent AuNPs-PEG_2000_-Nap 1 and 100% would support the same conditions. In this study, their colloidal stability was assessed using different methodologies, including UV–visible spectroscopy, DLS, and TEM (Table 2).

**Table 2:** Ultrastability characterization of **(A)** AuNPs-PEG_2000_-Nap 1% and **(B)** AuNPs-PEG_2000_-Nap 100% before treatment and after three cycles of 24-h freeze-drying, sterilization by an autoclave, three precipitations by ultracentrifugation, and three periods of 12-h heating at 65°C.

A	Before	Freeze-drying	Sterilization	Centrifugation	Heat
UV–visible	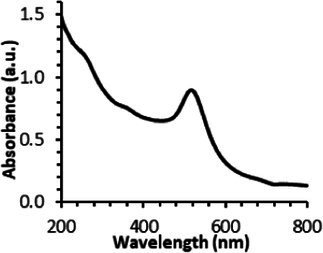	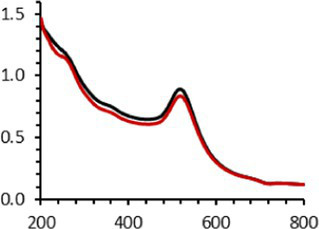	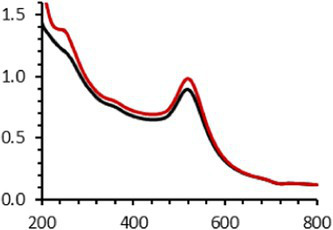	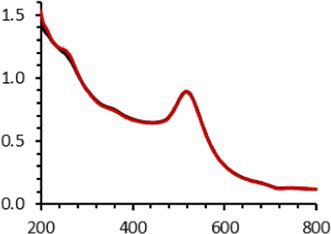	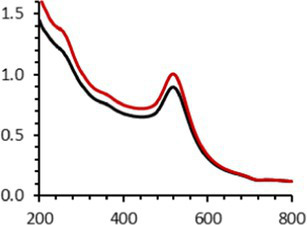
TEM (diameter)	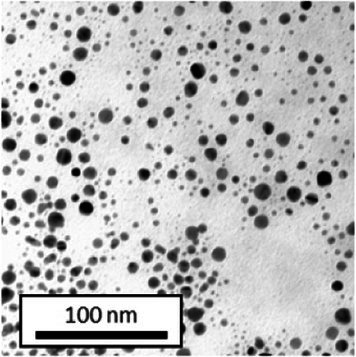 (6.0 ± 3.0) nm	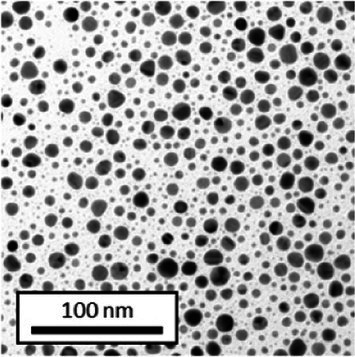 (6.4 ± 2.4) nm	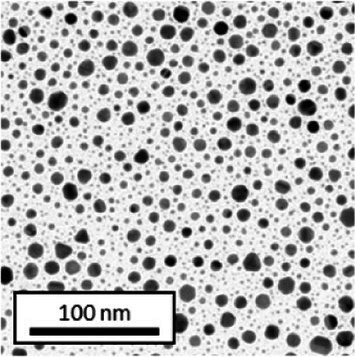 (8.2 ± 3.0) nm	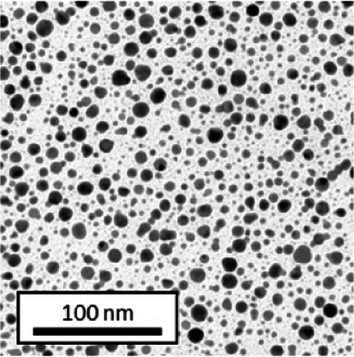 (5.4 ± 3.0) nm	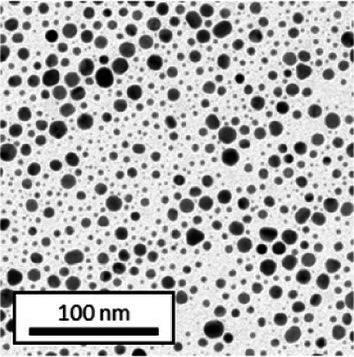 (6.2 ± 2.8) nm
DLS (diameter)	(29.0 ± 1.0) nm	(28.6 ± 0.6) nm	(27.8 ± 0.4) nm	(26.8 ± 0.8) nm	(28.0 ± 0.6) nm
B	Before	Freeze-drying	Sterilization	Centrifugation	Heat
UV–visible	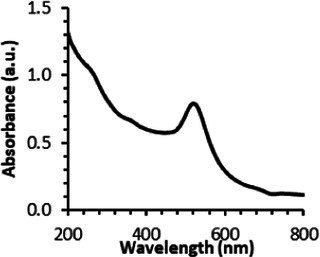	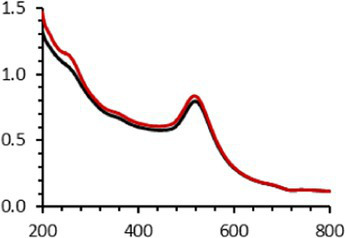	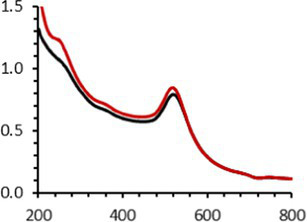	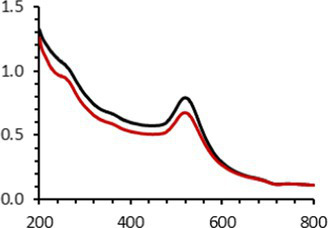	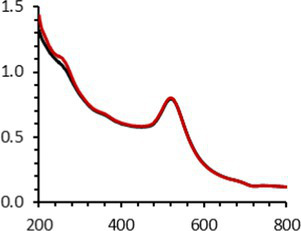
TEM (diameter)	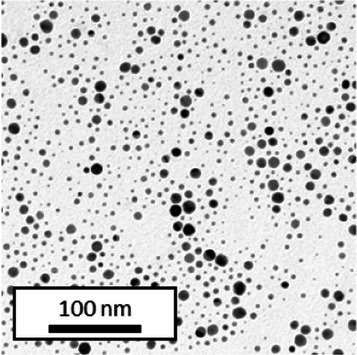 (4.8 ± 2.4) nm	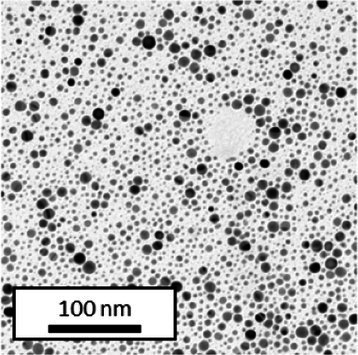 (4.0 ± 2.6) nm	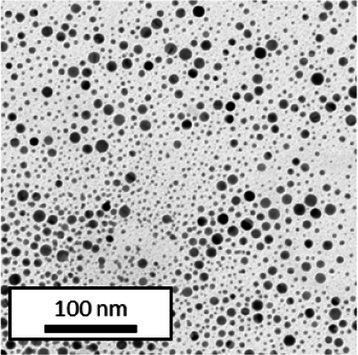 (4.8 ± 2.6) nm	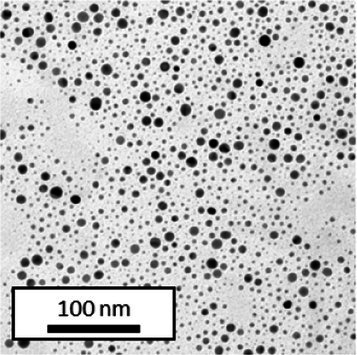 (4.2 ± 2.4) nm	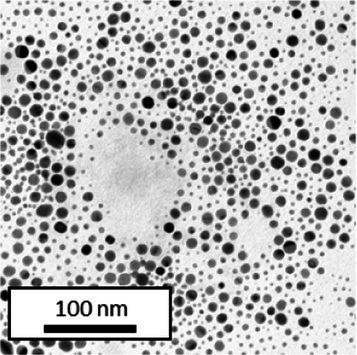 (4.6 ± 2.4) nm
DLS (diameter)	(34.0 ± 0.6) nm	(332 ± 74) nm	(37.4 ± 1.6) nm	(42.2 ± 1.6) nm	(39.4 ± 1.8) nm

The UV–visible spectra of the AuNPs-PEG2000-Nap before and after the different treatments are represented by the solid black and solid red lines, respectively. The core diameters and hydrodynamic diameters were obtained from the TEM images and DLS, respectively. The experimental details are given in the Materials and Methods.

An interesting characteristic of AuNPs is the ability of the conduction electrons of the gold atoms to oscillate coherently when irradiated by the oscillating electric field of light. The localized surface plasmon resonance (LSPR) effect causes an absorbance band in the electromagnetic spectrum, typically in the visible light range. The absorption peak can be altered by variations in different factors, e.g., the particle size and shape, coating, the distance between particles, pH, temperature, the number and types of the ligands linked to the gold core, and the surrounding physicochemical environment (136, 137). Here, the plasmonic band shift, which was measured accurately with the first derivative, was used to determine the colloidal stability of AuNPs-PEG_2000_-Nap 1 and 100% to different treatments (Supplementary Figure 3) (98). The colloidal stability results for AuNPs-PEG_2000_-Nap 1 and 100% are shown in Table 2.

The UV–visible spectra were obtained with 0.050 mg/mL of AuNPs-PEG_2000_-Nap 1 and 100% before and after three cycles of 24-h freeze-drying, three periods of 12-h heating at 65°C, and three precipitations by ultracentrifugation, and sterilization using an autoclave (Table 2). Tables 2A,B show the colloidal stability results for AuNPs-PEG_2000_-Nap 1% and AuNPs-PEG_2000_-Nap 100%, respectively. As can be seen, the UV–visible spectrum for each AuNP-PEG_2000_-Nap observed after treatment, represented by the full red lines, is nearly the same as the initial spectrum, represented by the full black lines. The LSPR position of AuNPs-PEG_2000_-Nap 1% before and after all treatments, which was determined by calculating the derivative of the spectra, remained at 517.5 nm (Supplementary Figure 1). For AuNPs-PEG_2000_-Nap 100%, the derivative of the plasmon bands exhibited a slight shift, translating into a disturbance of the gold core. Specifically, only the sterilization caused a 0.75 nm shift of the plasmon band peak of AuNPs-PEG_2000_-Nap 1%, and it caused a 2.00 nm shift for AuNPs-PEG_2000_-Nap 100% (Supplementary Figure 4).

AuNPs-PEG_2000_-Nap 1% were able to sustain the ultrastability testing as very small variations in the UV–visible spectra, the TEM images, and the hydrodynamic diameters can be observed. AuNPs-PEG_2000_-Nap 100% also exhibited little variation in the physicochemical parameters after freeze-drying, centrifugation, sterilization, and heating. In addition, the plasmon bands observed in the UV–visible spectroscopy remained similar with no variations in the observed plasmon band peak position. However, a slight increase in the intensity of the entire spectrum of AuNPs-PEG_2000_-Nap 1% was observed after sterilization and heating, which is likely due to the evaporation of a small part of the total volume. For AuNPs-PEG_2000_-Nap 100%, a slight reduction in the global intensity was observed after centrifugation, which suggests that some of the AuNPs-PEG_2000_-Nap 100% could aggregate during this step. The mean core of the diameter of AuNPs-PEG_2000_-Nap 1 and 100%, extracted from TEM images using the ImageJ software, did not vary significantly after each treatment (Table 2). The DLS experiments yielded quite similar results after all treatments and showed no signs of aggregation for AuNPs-PEG_2000_-Nap 1%. However, the same cannot be said for AuNPs-PEG_2000_-Nap 100%. Here, the hydrodynamic diameter varied significantly depending on the treatment, especially after freeze-drying (from 34.0 ± 0.6 nm to 332 ± 74 nm, as shown in Table 2B). The increased number of Nap groups appeared to alter the colloidal stability, thereby leading to a likely agglomeration in the solution. Note that a detectable level of fluorescence was maintained after all treatments for both AuNPs (Supplementary Figure 5).

To the best of our knowledge, the ultrastable AuNPs-PEG_2000_-Nap 1% are the first reported fluorescent AuNPs that can sustain autoclave sterilization without major changes in their UV–visible spectra and both the core and hydrodynamic diameters, as previously showcased with their nonfluorescent counterpart (90). As mentioned previously, colloidal stability is essential to preserve the functionality of AuNPs-PEG_2000_ and AuNPs-PEG_2000_-Nap, especially if AuNPs-PEG_2000_ are used as a drug delivery system for topically applied treatments (102). The results of the physical characterization suggest that AuNPs-PEG_2000_-Nap 1% could be a promising candidate as an imaging tool for AuNPs-PEG_2000_. Combined with the ultrastability assay, the previous fluorescence experiments (Figure 1) have both shown that AuNPs-PEG_2000_-Nap 1% is the most suitable fluorescent probe for imaging purposes while offering the same properties as the nonfluorescent AuNPs-PEG_2000_ (90). Thus, we focus on AuNPs-PEG_2000_-Nap 1% in all subsequent experiments.

#### 3.2.2 Mucoadhesion

Various techniques have been developed to study mucoadhesion in order to quantify and characterize how different materials interact with mucins (81, 138, 139). Mucins are glycoproteins found in the airways, gastrointestinal tract, genitourinary tract, nasal cavity, mouth, throat, and ocular surface (140). As the bottom layer of the tear film, the mucoid layer on the ocular surface is responsible for the maintenance of the lacrimal fluid, lubrication to facilitate blinking, ensuring a smooth surface for vision, and protection by trapping and removing pathogens and debris (141). Mucoadhesive drug delivery systems can increase their residence time in the precorneal film, which could broaden the drug release window and potentially reduce the required number of eyedrop applications (142).

A protocol adapted for AuNPs mucoadhesion involves the periodic acid/Schiff’s reagent (PAS) coloration method (143). This method analyzes the mucin content and mucoadhesion by oxidizing saccharide hydroxyl groups and reacting them with decolored fuchsin, resulting in a color change that is detectable by UV–visible spectroscopy (144). A protocol has been perfected and optimized for its combination with AuNPs, in consideration of their high visible light absorbance (108).

**Figure 2:**
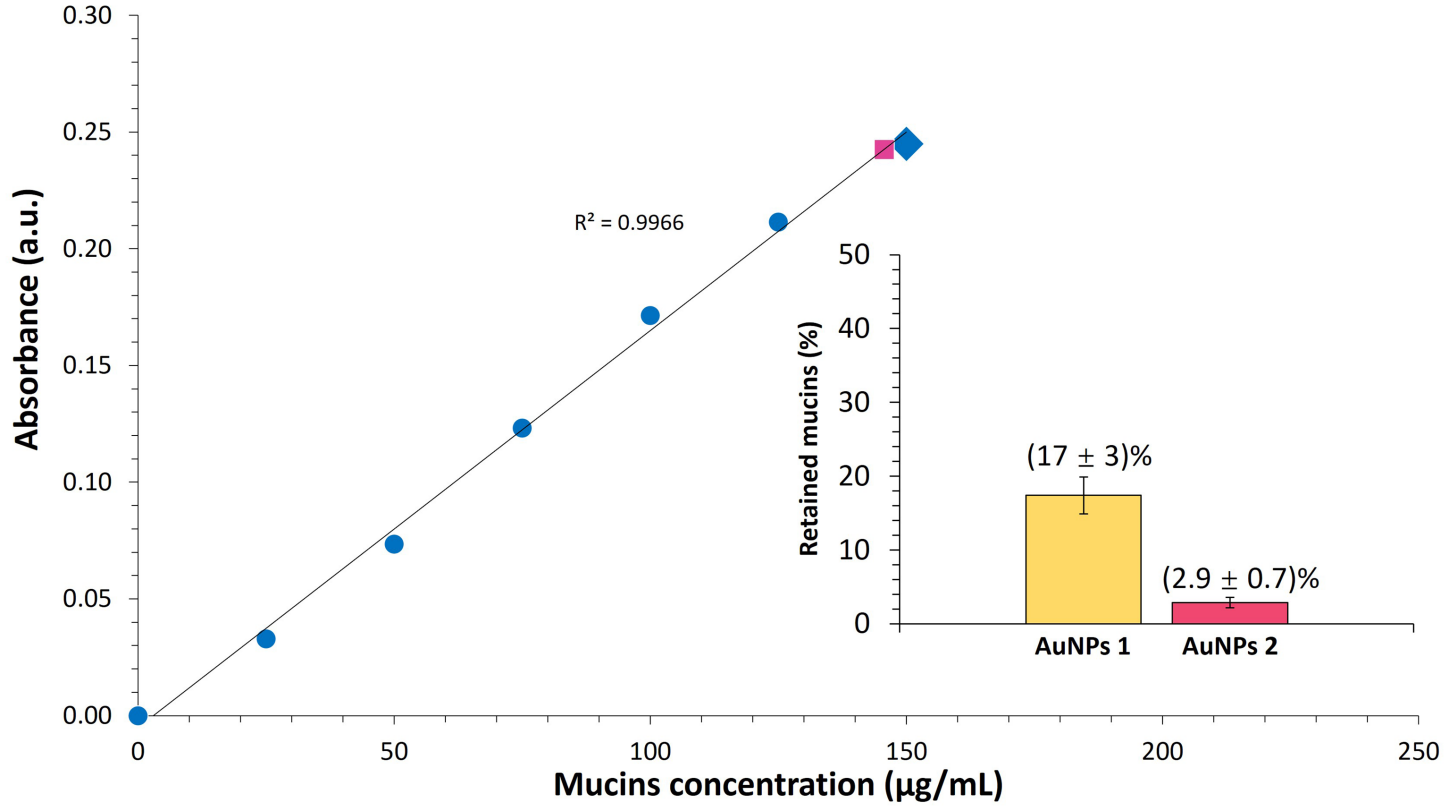
Quantitative analysis of mucins adsorbed on AuNPs. The calibration curve of the mucins is represented by the blue circles. The yellow triangle represents the absorbance coming from the concentration of mucins still free after the mix between the AuNPs (1 mg/mL) with an initial mucin concentration of 150 μg/mL (large blue diamond), and the pink square represents mucins still free after contact with AuNPs-PEG_2000_-Nap 1% (1 mg/mL). Insert: Histogram of the percentage of adsorbed mucins on AuNPs-PEG_2000_ (AuNPs 1) and AuNPs-PEG_2000_-Nap 1% (AuNPs 2). The data are reported as the mean ± standard deviation (*n* = 3).

The nonfluorescent AuNPs-PEG_2000_ could adsorb 11 ± 4% of the 150 μg of mucins they were incubated with, and the AuNPs-PEG_2000_-Nap 1% were able to retain 2.9 ± 0.7% of the same initial amount of mucins (Figure 2). AuNPs can exhibit mucoadhesive properties due to their ability to bind to mucins through two main mechanisms, i.e., (1) the formation of S-S bonds with the thiol groups at the metallic core and (2) the direct interaction of the cysteine groups with the gold core via Au-S bonds. The presence of some peripheral Nap groups on AuNPs-PEG_2000_-Nap 1% could account for the observed differences compared with conventional AuNPs-PEG_2000_. When mucins attempt to interact near the core, the steric hindrance from the Nap groups may prevent the access of many proteins. In addition, the very high graft density (3.05 ligands/nm^2^) of PEGylated ligands on AuNPs-PEG_2000_-Nap 1% could contribute to the high motility in mucus (145). In fact, the AuNPs-PEG_2000_ and AuNPs-PEG_2000_-Nap 1% exhibited low percentages of adsorbed mucins; however, their high draft density could increase the diffusion into the mucous layer. This could potentially increase the residence time of the drug delivery systems, which would increase the local concentrations of therapeutic molecules near the corneal epithelial cells.

#### 3.2.3 Cytotoxicity

**Figure 3:**
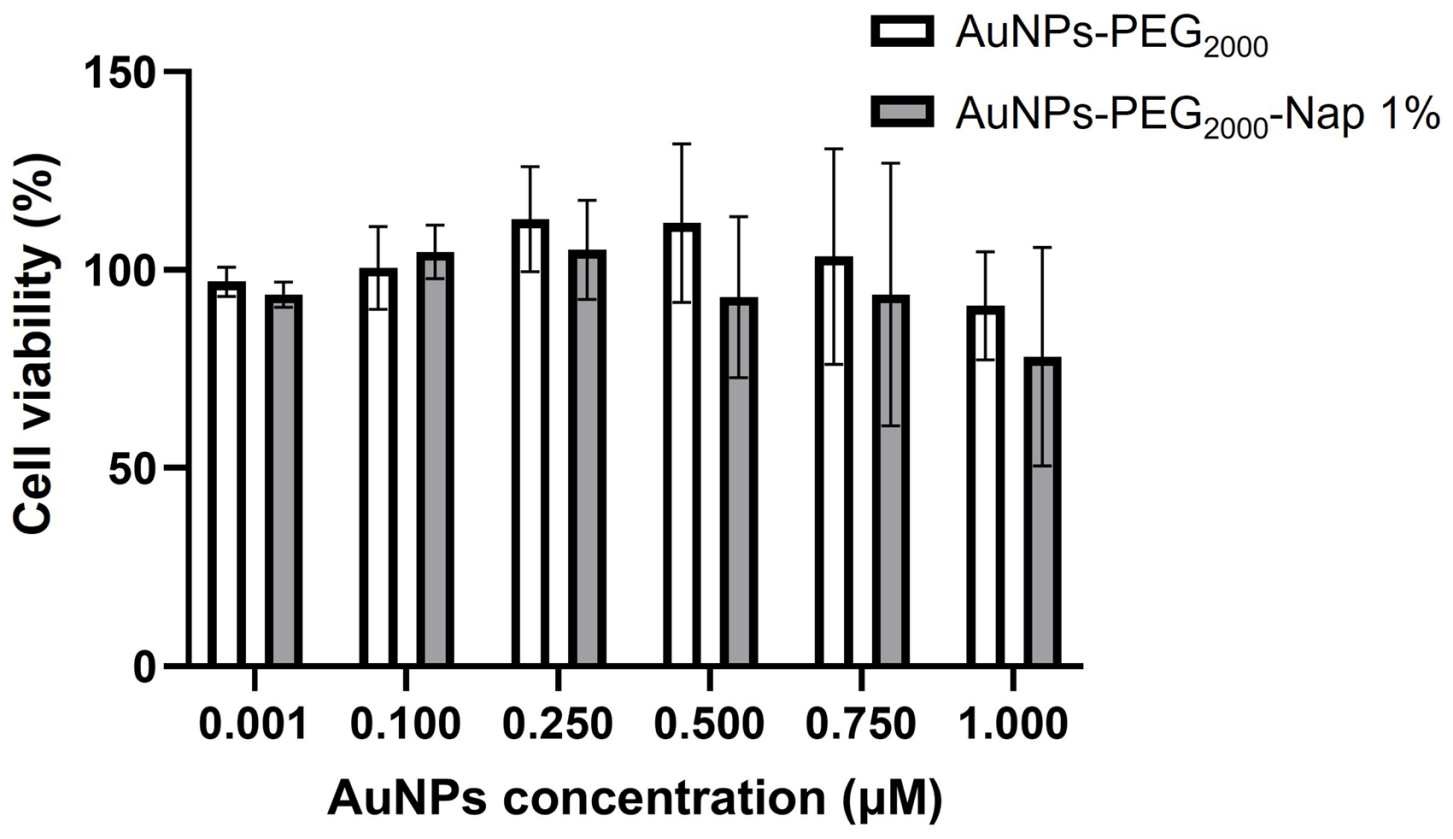
Effect AuNPs-PEG_2000_ and AuNPs-PEG_2000_-Nap 1% on cell viability. The hCECs were cultured as monolayers (10,000 cells per well, three wells per condition, repeated using three different populations) and exposed to increasing doses of AuNPs-PEG_2000_ and AuNPs-PEG_2000_-Nap 1% for 18 h. Cell viability was assessed using an MTS assay. Results are presented as mean ± standard deviation.

In a previous paper, MTS viability assays with different types of AuNPs, including AuNPs-PEG_2000_, were performed at low AuNP concentrations (from 0.0005 to 0.406 μM) (90), and showed a high cell viability for all concentrations tested. Herein, a MTS viability assay was conducted to determine if the addition of the Nap group to the AuNPs-PEG_2000_ could impact cell viability. We also increased the range of AuNP concentrations to evaluate the highest concentration that could affect cell viability. Results for each AuNP concentration and type (Figure 3) show a similar viability between the AuNPs-PEG_2000_ and the AuNPs-PEG_2000_-Nap 1% for all the concentrations tested, meaning that fluorescent molecules covalently bonded to PEG do not impact biological response differently that the non-fluorescent ones (only descriptive statistics were used). Furthermore, results show that concentrations between 0,0001 and 0,75 μM AuNPs had a cell viability similar to the control (no AuNPs), and only a 9 ± 14 and a 22 ± 28% cell death was observed for the highest concentration of AuNPs (1 μM) for, respectively the AuNPs-PEG_2000_ and the AuNPs-PEG_2000_-Nap 1%. These results indicate that AuNPs-PEG_2000_-Nap 1% are adequate for fluorescent biolocalization studies and biodistribution experiments.

#### 3.2.4 Cell internalization

**Figure 4:**
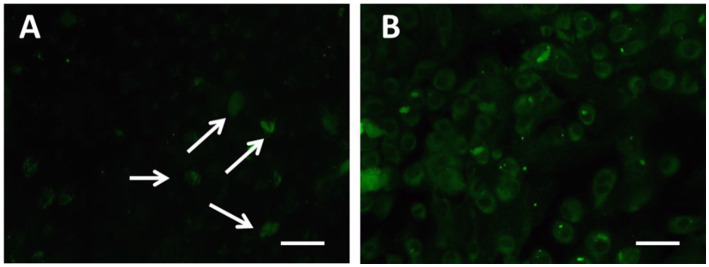
Fluorescence assay of internalized of **(A)** control AuNPs-PEG_2000_ and **(B)** AuNPs-PEG_2000_-Nap 1% (green) by cultured hCECs with FITC filters. The arrows point to hCECs slightly showing autofluorescence. Scale bars: 10 μm.

Finally, prior to performing *ex vivo* localization experiments, AuNPs-PEG_2000_ and AuNPs-PEG_2000_-Nap 1% were placed into wells of hCECs for 30 min to gather data on possible AuNPs internalization into the cells. After incubation for 30 min, the wells were rinsed with a phosphate-buffered saline (PBS) wash and then observed under an epifluorescence microscope using the fluorescein isothiocyanate (FITC) filters. The results indicate that some hCECs exhibit a small degree of autofluorescence in the same wavelength range as the AuNPs-PEG_2000_-Nap 1% emission (Figure 4A). However, the distinction between this background fluorescence and the emitted fluorescence coming from AuNPs-PEG_2000_-Nap 1% observed in Figure 4 is obvious because the cells are clearly visible in Figure 4B compared with Figure 4A. In addition, this assay confirmed that AuNPs-PEG_2000_-Nap 1% are internalized into hCECs because fluorescence can be observed in the cytoplasm and is absent in the nucleus. If the AuNPs-PEG_2000_-Nap 1% had covered the surface of the cell without penetrating the cells, the fluorescence would have been uniform, and a distinction between the cytoplasm and nucleus would not be observable.

Thus, the AuNPs-PEG_2000_-Nap 1% emit strong fluorescence with minimal change to the original nonfluorescent AuNPs-PEG_2000_ by simply adding a fluorescent probe to 1% of their attached PEGylated ligands (approximately 3–4 Nap groups per gold core). In addition, they exhibit the same ultrastability properties as the AuNPs-PEG_2000_, i.e., they can sustain rigorous conditions without significantly impacting the plasmon band position, the colloidal stability measured by DLS, and the gold core diameter measured using TEM images. Furthermore, compared to the AuNPs-PEG_2000_, addition of the PEG_2000_-Nap 1% did not reduce cell viability, as shown by the results of the MTS assay (Figure 3). Combined, these similarities demonstrate that AuNPs-PEG2000-Nap 1% can be utilized as a substitute for AuNPs-PEG2000 to study their biodistribution and biolocalization.

### 3.3 Biolocalization study using AuNPs-PEG_2000_-nap 1%

A significant challenge in clinical translation lies in the lack of animal models that accurately replicate the human ocular system’s anatomy and physiology. Rodents are commonly used for safety and efficacy evaluations due to their cost-effectiveness and ease of handling. However, the extrapolation of pharmacodynamic responses from rodents to humans can be unreliable due to significantly different anatomy (146). Compared to rodents, rabbits exhibit greater anatomical and physiological similarities to humans, particularly regarding eye size, vitreous humor volume, and internal structure, providing a comparable pathway for topically administered compounds (147). Nevertheless, key differences remain, such as the higher viscosity of rabbit aqueous humor, larger anterior chambers, and greater blinking frequency, which must be carefully considered (148). In this study, *ex vivo* rabbit eyeballs were used, thereby eliminating significant factors impacting biodistribution and elimination of topically applied nanoparticles, such as tear film dynamics, blinking and systemic circulation (149, 150). Whole eyeballs from slaughterhouse rabbits were used in the biolocalization experiments. Here, a drop (50 μL, 1 mg/mL) of AuNPs-PEG_2000_-Nap 1% diluted in PBS was placed on the cornea for each eyeball. The application time was either 3 min to replicate the renewal rate of the tear film (9) or 2 h to increase the interaction probability with the tear film and their diffusion. In addition, the corneas, irises, lenses and posterior segments were dissected carefully to avoid contamination between the different ocular tissues and then embedded in an optimum cutting temperature (OCT) compound and cut at the cryostat. No fixation steps were carried out before microscopy in order to avoid any physicochemical modification of the eye tissues that could impact the localization of AuNPs-PEG_2000_-Nap 1%. Therefore, this method provides a more direct way to observe the biodistribution of gold nanoparticles in the different structures of the eye (151).

#### 3.3.1 Cornea

The human cornea comprises three cellular layers (i.e., the epithelium, stroma, and endothelium) and two acellular layers that separate them (i.e., Bowman’s layer and Descemet’s membrane) (152).

**Figure 5:**
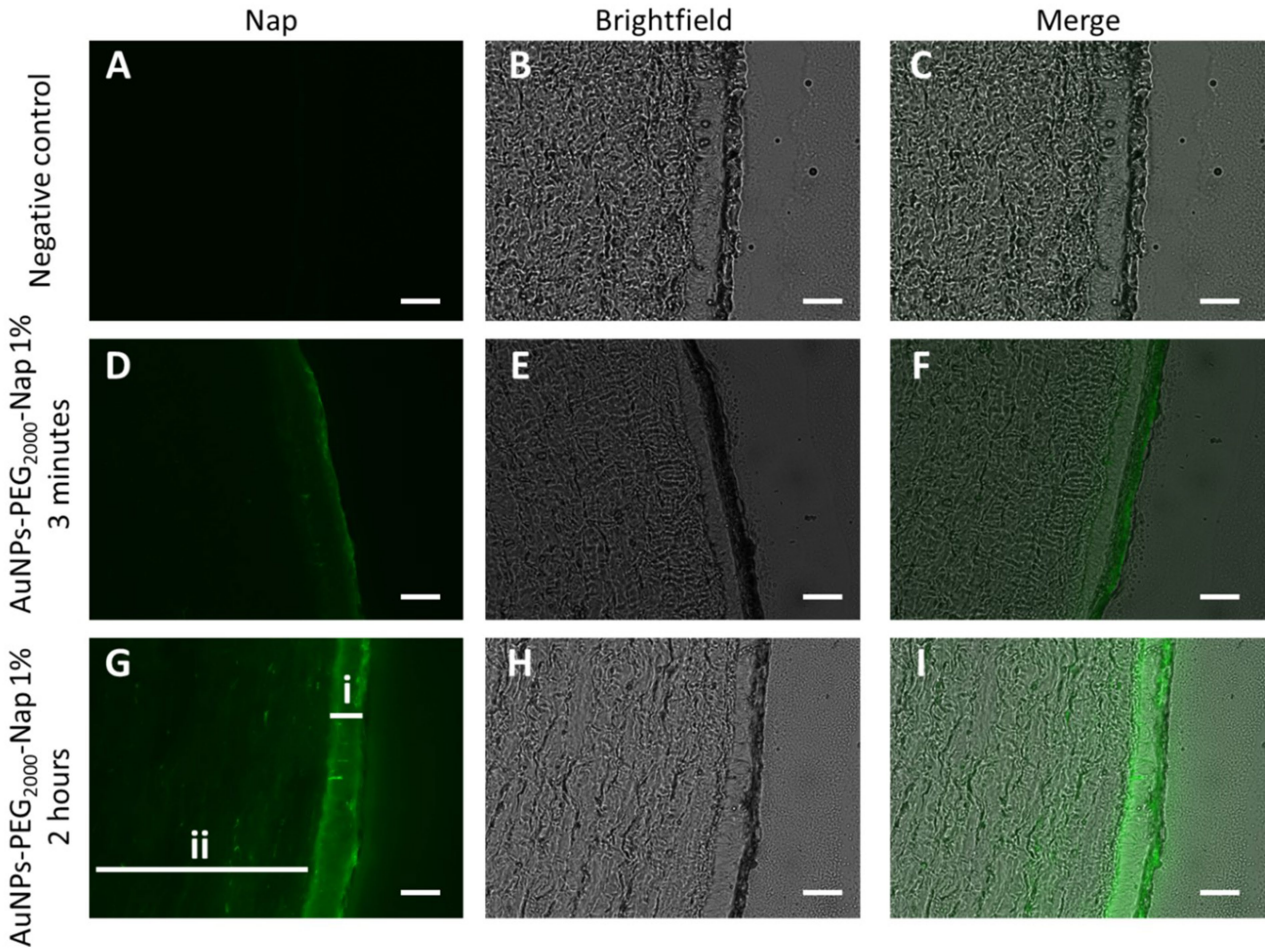
Localization of AuNPs-PEG_2000_-Nap 1% (green) in rabbit cornea cross-sections observed under a fluorescence microscope using the FITC filter (first column), brightfield (middle column), and merge of the two (third column). Images of the cornea **(A)**, **(B)**, and **(C)** after 2-h PBS application; **(D)**, **(E)**, and **(F)** 3-min application of AuNPs-PEG_2000_-Nap 1% followed by a 2-h wait; and **(G)**, **(H)**, and **(I)** 2-h application of AuNPs-PEG_2000_-Nap 1%, where i is the multistratified epithelium, and ii is the stroma. Scale bars: 50 μm.

AuNPs-PEG_2000_-Nap 1% were found in the cornea for both application times (Figure 5). Note that more AuNPs-PEG_2000_-Nap 1% were found in the corneal epithelium after 2 h, as the epithelial layer was more defined, clearly observable, and brighter, as shown in Figure 5G. The 3-min application was sufficient for AuNPs-PEG_2000_-Nap 1% to adhere to the ocular surface because fluorescence can be observed in the corneal epithelium (Figure 5D) even after the vigorous PBS wash. Fluorescent AuNPs-PEG_2000_-Nap 1% were also found in the stroma for both application times, which means that the AuNPs-PEG_2000_-Nap 1% were not confined to the direct application site or the corneal epithelium, benefiting motility by their high graft density. In addition, there appeared to be an affinity between AuNPs-PEG_2000_-Nap 1% and the corneal epithelium because the brightness levels between the epithelial layer and the stroma present a clear intensity difference, especially after 2 h.

The AuNPs-PEG_2000_-Nap 1% are not the first AuNPs to be topically applied and found in the corneal epithelium and stroma (77, 79, 80) but they are the only PEGylated AuNPs synthesized from a one-pot synthesis.

#### 3.3.2 Iris

The iris plays a crucial role in visual function by regulating the amount of light entering the eye and reaching the retina (153). The iris also participates in the circulation of aqueous humor, thereby helping to regulate the intraocular pressure (154).

**Figure 6:**
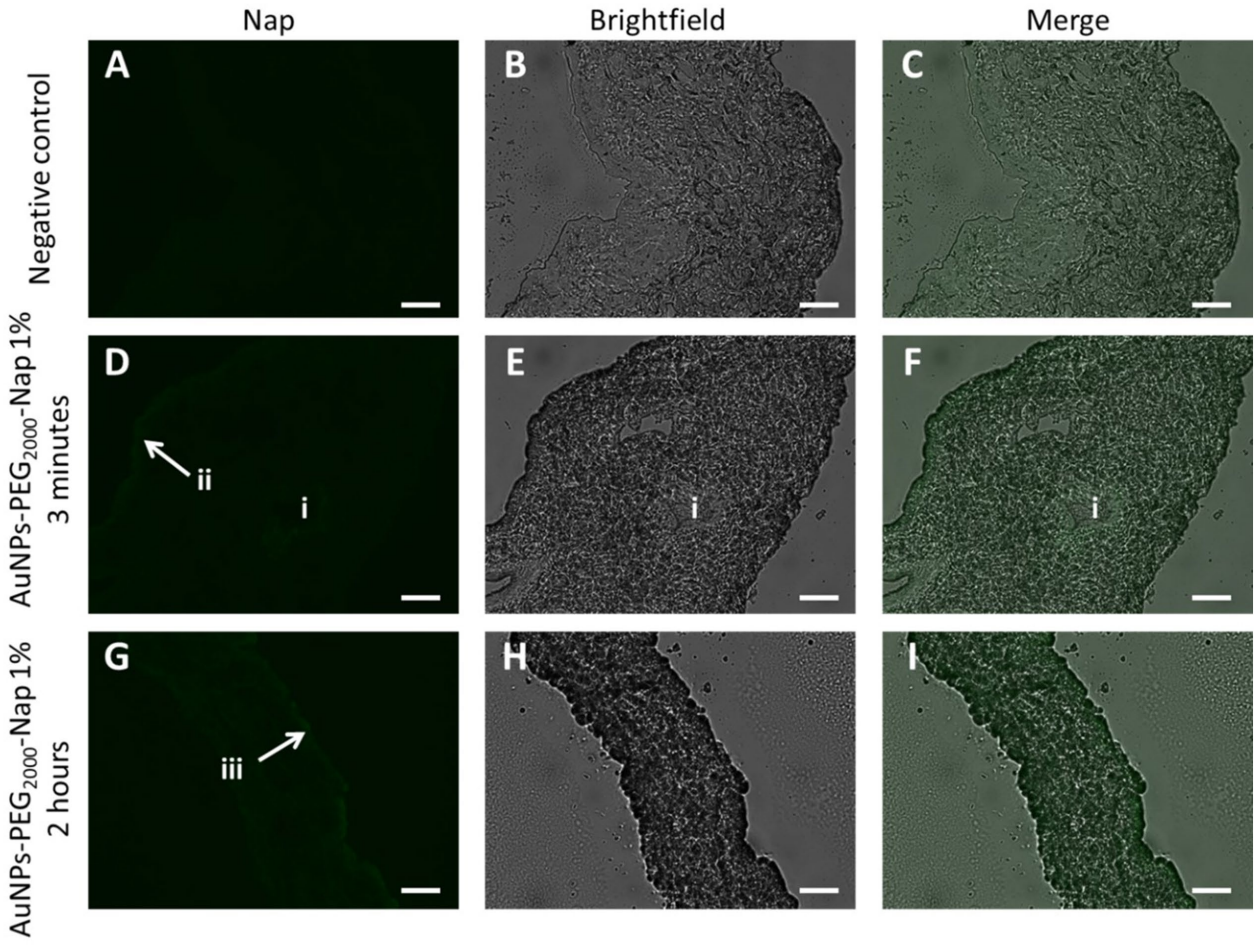
Localization of AuNPs-PEG_2000_-Nap 1% (green) in rabbit iris cross-sections observed under a fluorescence microscope using the FITC filter (first column), brightfield (middle column), and the merge of the two (third column). Images of the iris **(A)**, **(B)**, and **(C)** after 2-h PBS application; **(D)**, **(E)**, and **(F)** 3-min application of AuNPs-PEG_2000_-Nap 1% followed by a 2-h wait; and **(G)**, **(H)**, and **(I)** 2-h application of AuNPs-PEG_2000_-Nap 1%, where i represents the lumen of a blood vessel, ii represents a peripheral region where AuNPs-PEG_2000_-Nap 1% can be seen, and iii shows AuNPs-PEG_2000_-Nap 1% accumulation in the outermost structure of the iris. Scale bars: 50 μm.

Fluorescence can be faintly observed in the Nap images (Figure 6) without any clear preference for an iris structure. For the image obtained after the 3-min application, the lumen of a blood vessel was visible, in addition to the outermost periphery of the structure (Figure 6D), and AuNPs-PEG_2000_-Nap 1% can be observed in the extremity of the iris for the 2-h application (Figure 6G).

Note that the experiments were conducted using eyes from albino rabbits, which possess no pigment; thus, the iris pigment epithelium and the pigmented anterior surface were difficult to identify. However, AuNPs-PEG_2000_-Nap 1% had a strong affinity with the corneal epithelium (Figure 5); thus, we can expect to find AuNPs in similar cell types in other tissues, e.g., the epithelium of the iris. Nevertheless, it is important to underline that it is impossible to discern with certitude the exact iris structure in which the AuNPs-PEG_2000_-Nap 1% were found, only that they were found in the iris after only a 3-min application.

#### 3.3.3 Lens

The lens, which is an elastic and transparent biconvex structure located in the posterior chamber, comprises four parts, i.e., the lens capsule, epithelial cells, lens fibers, and zonules (155).

**Figure 7:**
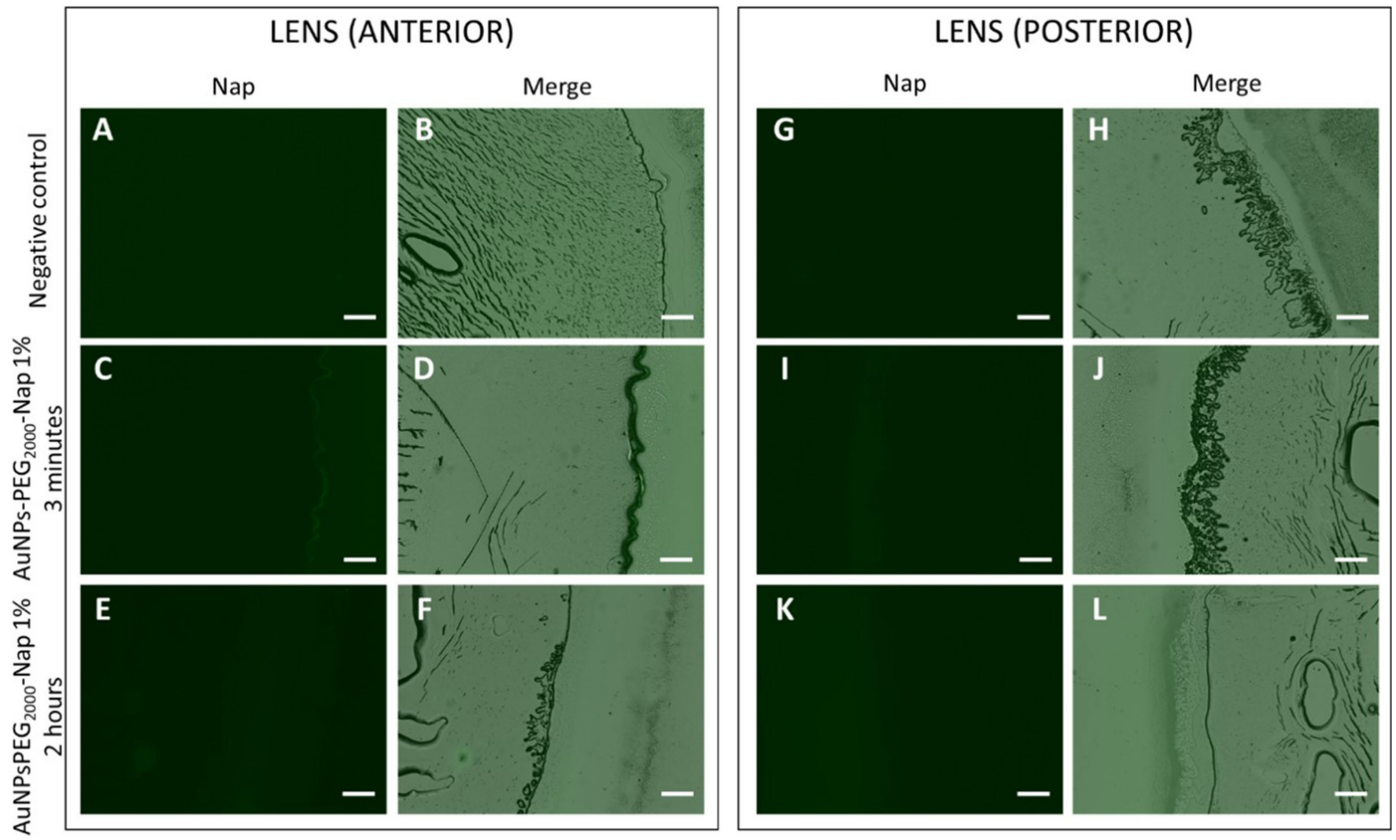
Localization of AuNPs-PEG_2000_-Nap 1% (green) in the rabbit lens cross-sections observed under a fluorescence microscope using the FITC filter (first column) and the merge of the brightfield and FITC filter (second column) divided in two parts, where the anterior segment is closest to the cornea, and the posterior segment is closest to the back of the eye. Images of the anterior part of the lens **(A)** and **(B)** after 2-h PBS application; **(C)** and **(D)** 3-min application of AuNPs-PEG_2000_-Nap 1% followed by a 2-h wait; and **(E)** and **(F)** 2-h application of AuNPs-PEG_2000_-Nap 1%; **(G)** and **(H)** images of the posterior part of the lens after 2-h PBS application; **(I)** and **(J)** 3-min application of AuNPs-PEG_2000_-Nap 1% followed by a 2-h wait, and **(K)** and **(L)** 2-h application of AuNPs-PEG_2000_-Nap 1%. Scale bar: 50 μm.

As shown in Figure 7, AuNPs-PEG_2000_-Nap 1% were observed in the anterior part of the lens after the 3-min application. In fact, the fluorescent probe accumulated slightly in the epithelial cell monolayer of the lens (Figure 7C). Note that the epithelium of the lens is only located on its anterior part. In the images of the posterior part of the lens (Figures 7G,L), no AuNPs-PEG_2000_-Nap 1% can be observed. Knowing this, the possibility of the AuNPs-PEG_2000_-Nap 1% being in the lens capsule rather than the lens epithelium is excluded because fluorescence would be visible on the posterior part of the lens. The same tendencies were observed for the 2-h application.

To conclude on the distribution of the AuNPs in the anterior segment of the eye, the AuNPs-Nap 1% were readily observed in the corneal epithelium, the iris, potentially in its pigment epithelium, and in the lens epithelium. The fluorescent probe successfully crossed the anterior part of the rabbits’ eyeballs; however, most of the fluorescence was observed in the cornea.

#### 3.3.4 Posterior segment

In these experiments, the sampled posterior segment comprised the retina, choroid, and sclera. The retina, which is located in the posterior part of the eye, is a transparent, light-sensitive tissue composed of multiple cellular layers (156). The retina includes the light-transducing neural retina and the retinal pigment epithelium (RPE) (157). The neural retina is a layered structure comprising six major types of neurons organized into three nuclear layers containing neuronal cell bodies (or somas) and two plexiform layers where synapses occur (158).

**Figure 8:**
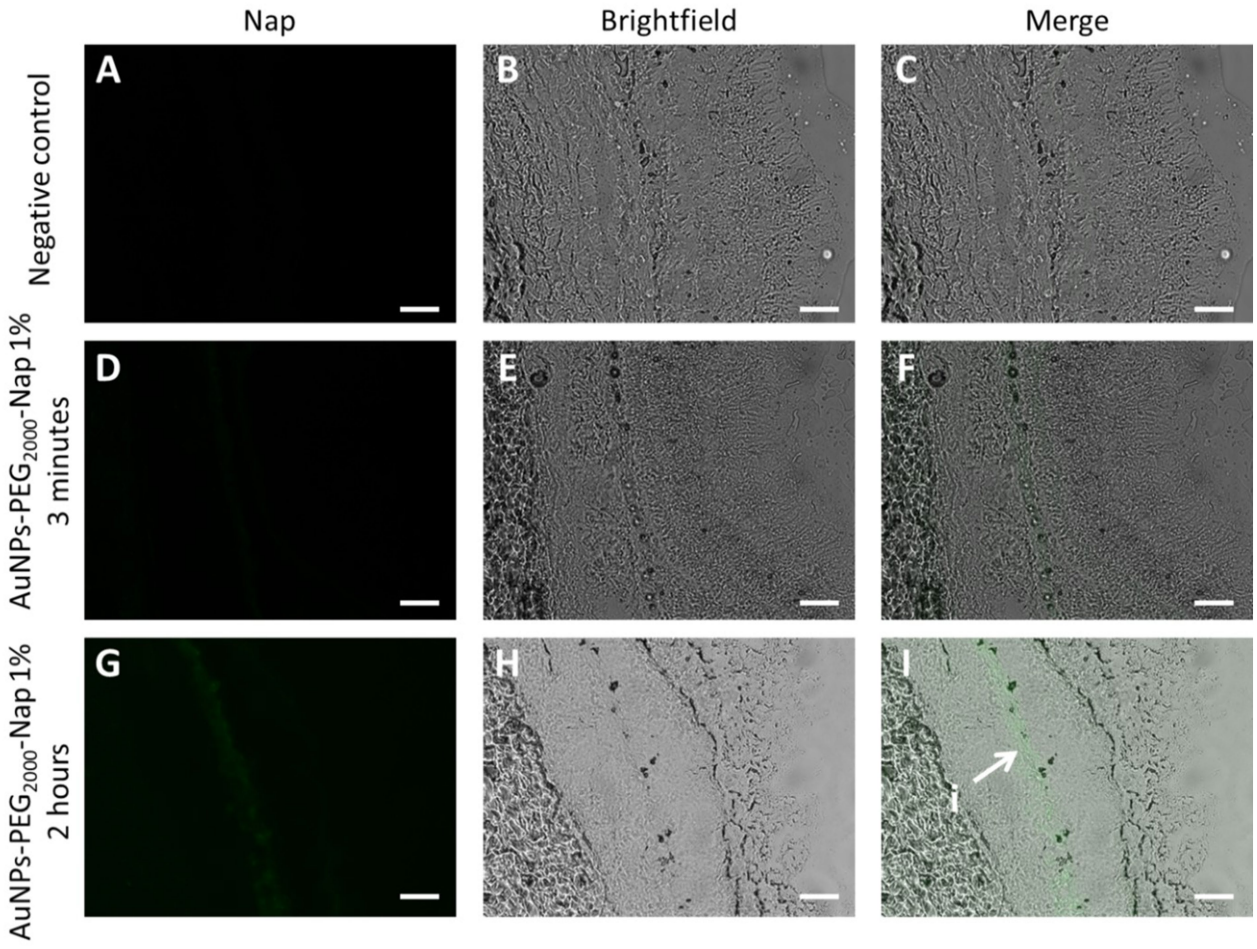
Localization of AuNPs-PEG_2000_-Nap 1% (green) in rabbit posterior segment cross-sections observed under a fluorescence microscope using the FITC filter (first column), brightfield (middle column), and merge of the two (third column). Images of the posterior segment after **(A)**, **(B)**, and **(C)** 2-h PBS application; **(D)**, **(E)**, and **(F)** 3-min application of AuNPs-PEG_2000_-Nap 1% followed by a 2-h wait; and **(G)**, **(H)**, and **(I)** 2-h application of AuNPs-PEG_2000_-Nap 1%, where i AuNPs-PEG_2000_-Nap 1% form a thin line. Scale bars: 50 μm.

Experimentally, a sample of the back of the eyeball was taken using a 10-mm diameter biopsy punch to facilitate manipulations. Here, the three-layering tissues, i.e., the retina, choroid, and sclera, were kept together. The AuNPs-PEG_2000_-Nap 1% were found in the posterior segment of the eye, as shown in Figure 8. For both application times, the AuNPs-PEG_2000_-Nap 1% were organized in a thin line in a specific structure of the retina; however, due to the lack of melanin pigment in albino rabbits, it is difficult to distinguish exactly where the choroid ends and the retina begins, thereby making it difficult to precisely identify the tissue sublayers where the AuNPs-PEG_2000_-Nap 1% are found. However, we did not expect to find them there because the AuNPs-PEG_2000_-Nap 1% had to go through the vitreous humor to migrate from the lens to the retina. The vitreous humor is a viscoelastic extracellular matrix hydrogel with a water content between 98 and 99.7% (159). The findings of this study demonstrate that the AuNPs-PEG_2000_-Nap 1% does not need to be injected via an intravitreous pathway to be found in the retina.

The AuNPs-PEG_2000_-Nap 1% are not the only AuNPs that were found in the retina after topical application (160). However, this phenomenon was previously demonstrated only in a mouse model, whose eye volume is approximately one hundred times smaller than that of a rabbit. Furthermore, *in vivo* biodistribution experiments benefit from having two possible routes for topically applied nanocarriers: the corneal and the non-corneal routes. Evidence also suggests that the non-corneal route is beneficial in bringing more nanocarriers into the posterior segment of the eye (104, 161–164). However, according to the design of our *ex vivo* experiments, it is impossible for the AuNPs-PEG_2000_-Nap 1% to take a route other than the corneal route. Thus, future *in vivo* experiments will be able to highlight the duality of corneal and non-corneal routes in addition to validating which route is preferable for PEGylated AuNPs.

Following the previous observations made in the anterior segment, where AuNPs-PEG_2000_-Nap 1% were frequently found in the corneal epithelium, observed in the iris, and successfully located and retained in the monolayer of the lens epithelium, it is possible that AuNPs-PEG_2000_-Nap 1% may be also found in the RPE. This structure is a monolayer of pigmented cells forming part of the blood-retina barrier (165), which is heavily involved in the transport of ions, water, and metabolic waste from the subretinal space to the blood (166, 167), as well as the transport of nutrients from the blood to the photoreceptors (168, 169). In addition, RPE cells perform specialized phagocytosis, which is crucial in terms of maintaining the health of photoreceptors by digesting their aged outer segments. This process prevents photo-oxidative damage to photoreceptors (170). RPE cells do not divide; thus, they must process the ingested material efficiently to avoid toxic buildup in their lysosomes, which can lead to retinal disorders (171). This could be the reason why the AuNPs-PEG_2000_-Nap 1% were concentrated in this region of the posterior segment. This creates an extremely dynamic environment where AuNPs-PEG_2000_-Nap 1% could potentially be found, fitting the description of tissues where they were observed in this study. However, further studies are required to investigate and confirm possibility.

## 4 Conclusion

In conclusion, AuNPs-PEG_2000_-Nap 1% were synthesized and exhibited similar properties as their nonfluorescent counterpart (AuNPs-PEG_2000_), e.g., ultrastability, low mucoadhesion, and low cytotoxicity, and they were internalized by the cells. When applied topically to *ex vivo* rabbit eyeballs, they demonstrated a strong affinity with the cornea, specifically the corneal epithelium. In addition, they were found in the iris, the lens epithelium, and the posterior segment of the eye. These results represent a tremendous opportunity for research on the potential of AuNPs as drug delivery systems.

The nanoparticle synthesis protocol developed in this study has the potential to be adapted for the creation of various types of AuNPs tailored to various applications and different target sites. This adaptability could be particularly beneficial for research focusing on the controlled release of active molecules in the cornea. By functionalizing the polymeric corona, it may be possible to increase mucoadhesion, thereby improving the delivery and retention of therapeutic agents (101).

Previously, nonfluorescent AuNPs-PEG_2000_ have demonstrated significant potential as drug carriers, particularly for delivering anti-inflammatory molecules (92). Building upon this foundation, future studies could investigate the encapsulation and release of active compounds designed to treat pathologies affecting the posterior segment of the eye. Such research could yield valuable insights and results, given that AuNPs can reach the back of the eye, thereby offering a promising delivery mechanism for drugs targeting this challenging area.

In this context, AuNPs have emerged as particularly promising candidates. For example, they have considerably potential for the targeted delivery of therapeutic molecules to the cornea, and they can reach the posterior segment of the eye. This dual functionality makes them an exciting prospect for future research, with the potential to advance the field of ocular drug delivery significantly. Thus, the continued investigation of their properties and applications could lead to new and more effective treatments for a range of eye conditions, benefiting patients with both anterior and posterior segment diseases.

## Data Availability

The original contributions presented in the study are included in the article/Supplementary material, further inquiries can be directed to the corresponding author.
