# Supplementary material for: Localization of fluorescent gold nanoparticles throughout the eye after topical administration
Source: Front Med (Lausanne). 2025 Mar 19;12:1557611. doi: 10.3389/fmed.2025.1557611 (PMC11961937; doi:10.3389/fmed.2025.1557611)
Supplement: Supplementary file 1 [file Data_Sheet_1.docx]

Supplementary Material


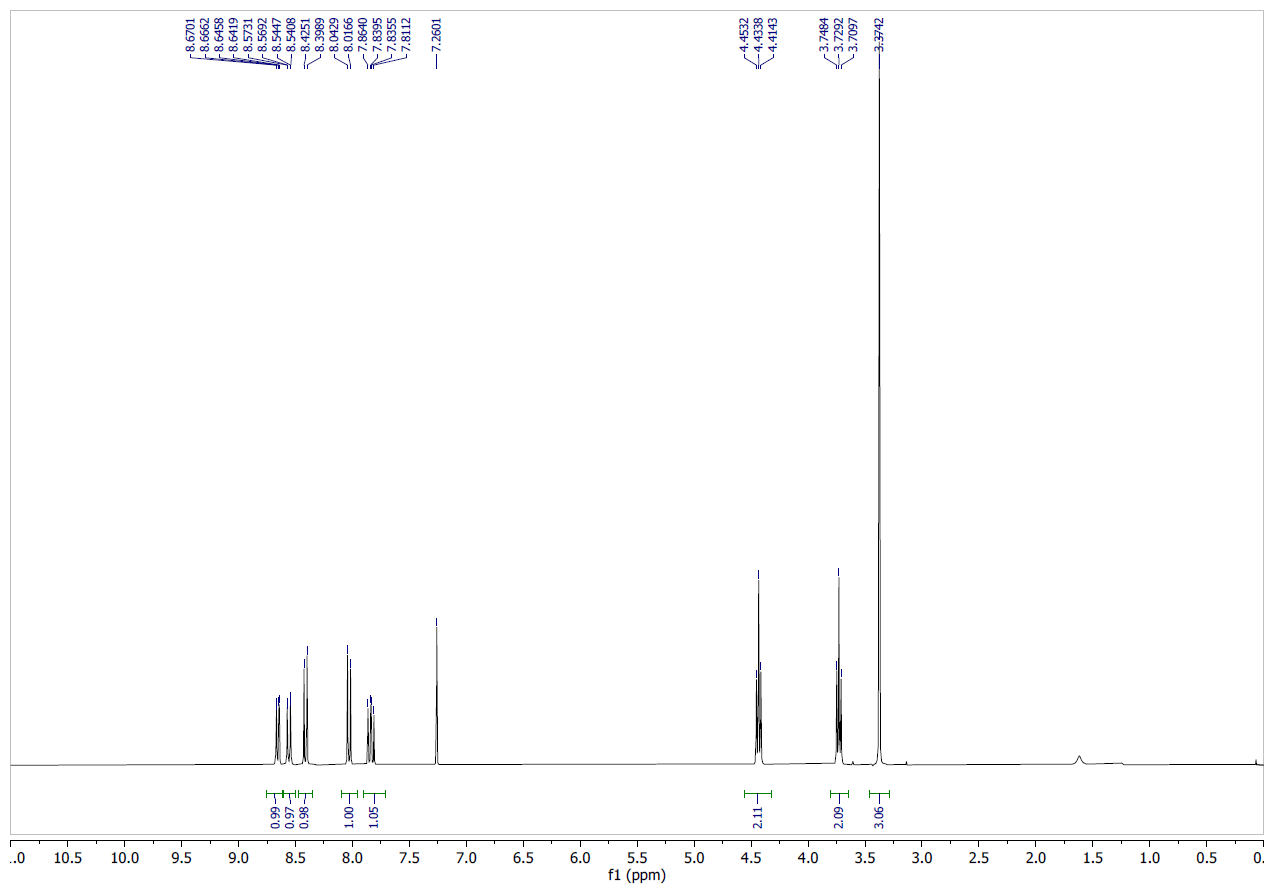
**Supplementary Figure 1.** ^1^H NMR spectrum of 4-bromo-N-(2-methoxyethyl)-1,8-naphthalimide in CDCl_3_ (300 MHz).


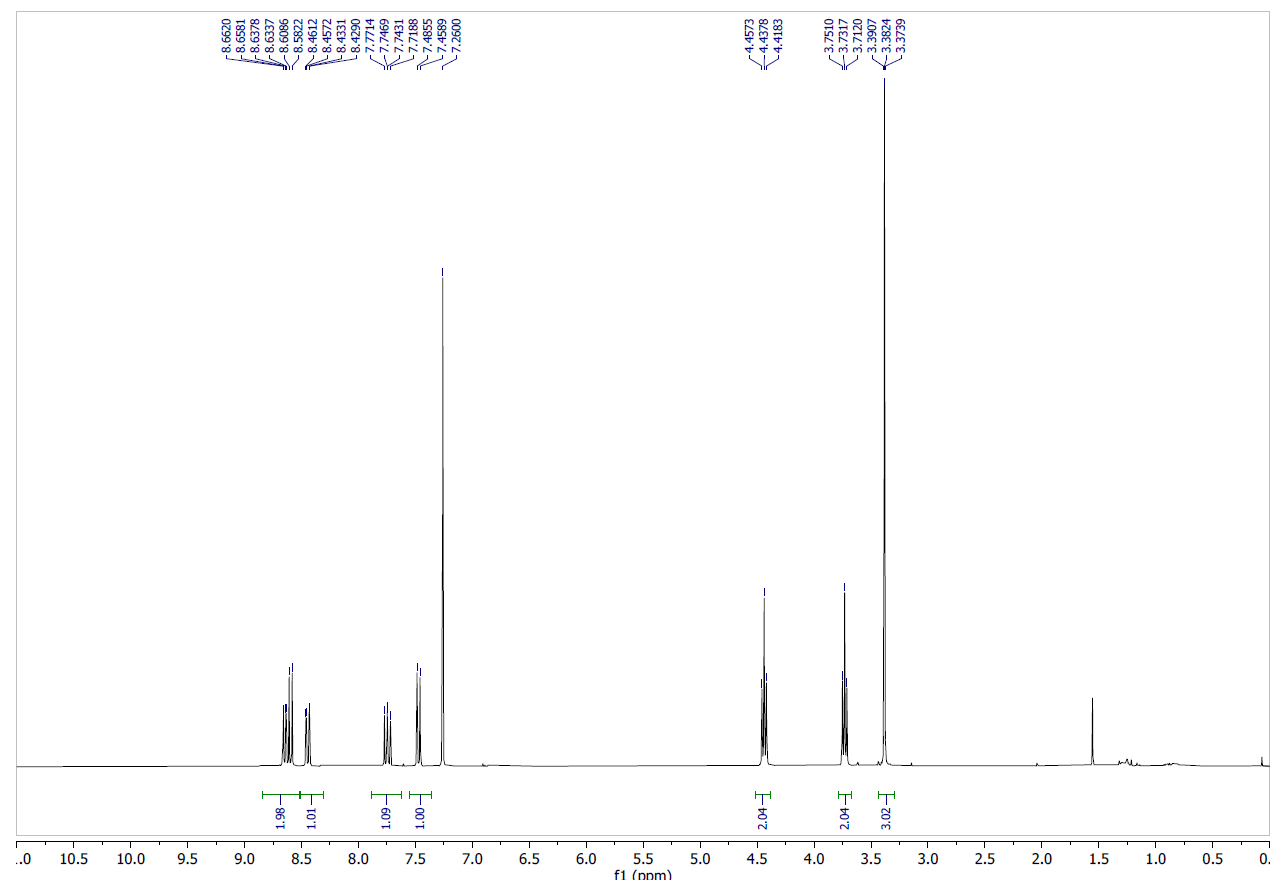


**Supplementary Figure 2.** ^1^H NMR spectrum of 4-azido-N-(2-methoxyethyl)-1,8-naphthalimide in CDCl_3_ (300 MHz).

**
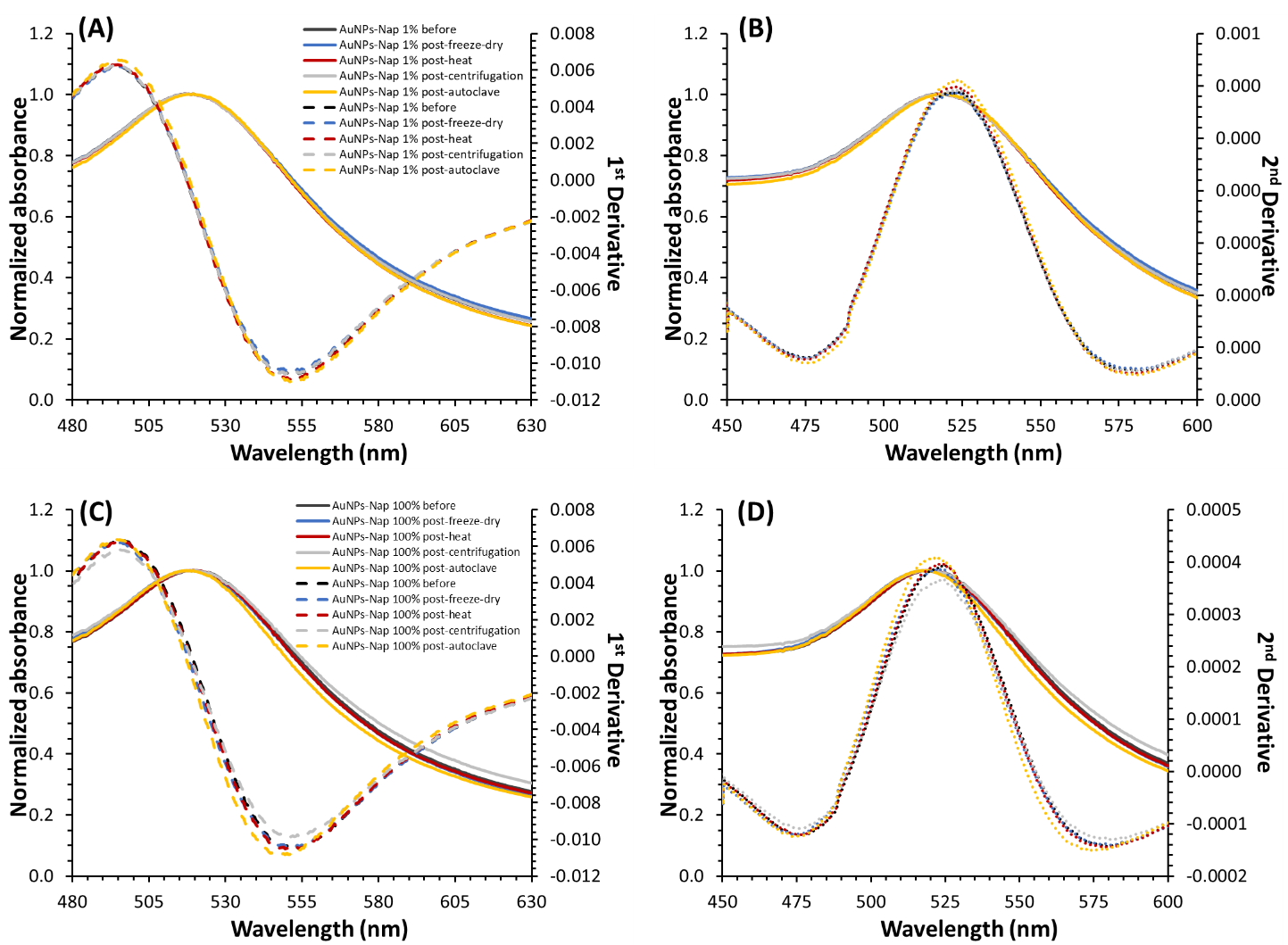
**

**Supplementary Figure 3.** UV-visible spectra of AuNPs-PEG_2000_-Nap 1% and AuNPs-PEG_2000_-Nap 100% before and after ultrastability assays in full lines. (A) The first derivative of the plasmon band of AuNPs-PEG_2000_-Nap 1% is represented by the dashed lines. The position of the plasmon band does not shift (517.50 nm) after freeze-drying, heating, and centrifugation; however, it shifts minimally after autoclaving (518.25 nm). (B) The second derivatives (dotted lines) of the AuNPs-PEG_2000_-Nap 1% plasmon band overlap, signaling a conserved form factor. (C) The first derivative of the plasmon band of AuNPs-PEG_2000_-Nap 100% (dashed lines) shows slight shifts of the plasmon band peak position (519.00 nm) after ultrastability assays (517.50 nm after freeze-drying, 518.5 nm after heating, 518.50 after centrifugation, and 517.00 nm after sterilization). (D) The second derivative of the plasmon band spectra of AuNPs-PEG_2000_-Nap 100% (dotted lines) do not overlap, translating into a loss of the form factor and thus a destabilization and modification of the environment near the gold core.


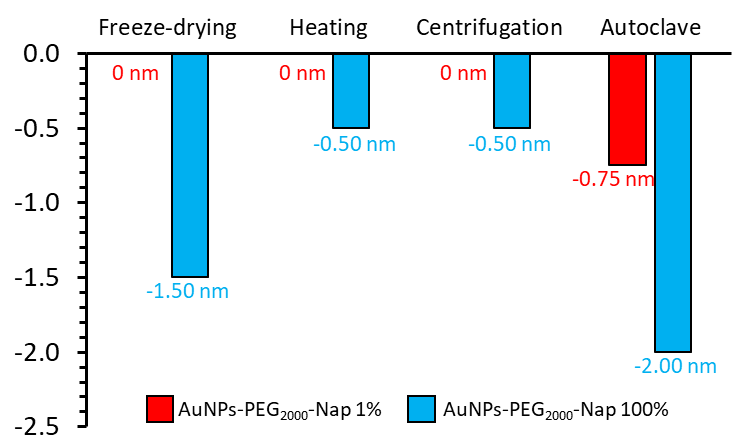


**Supplementary Figure 4.** Shifts of the plasmon band peak measured by the first derivative after the ultrastability assays compared with the position of the plasmon band prior to the ultrastability assay.


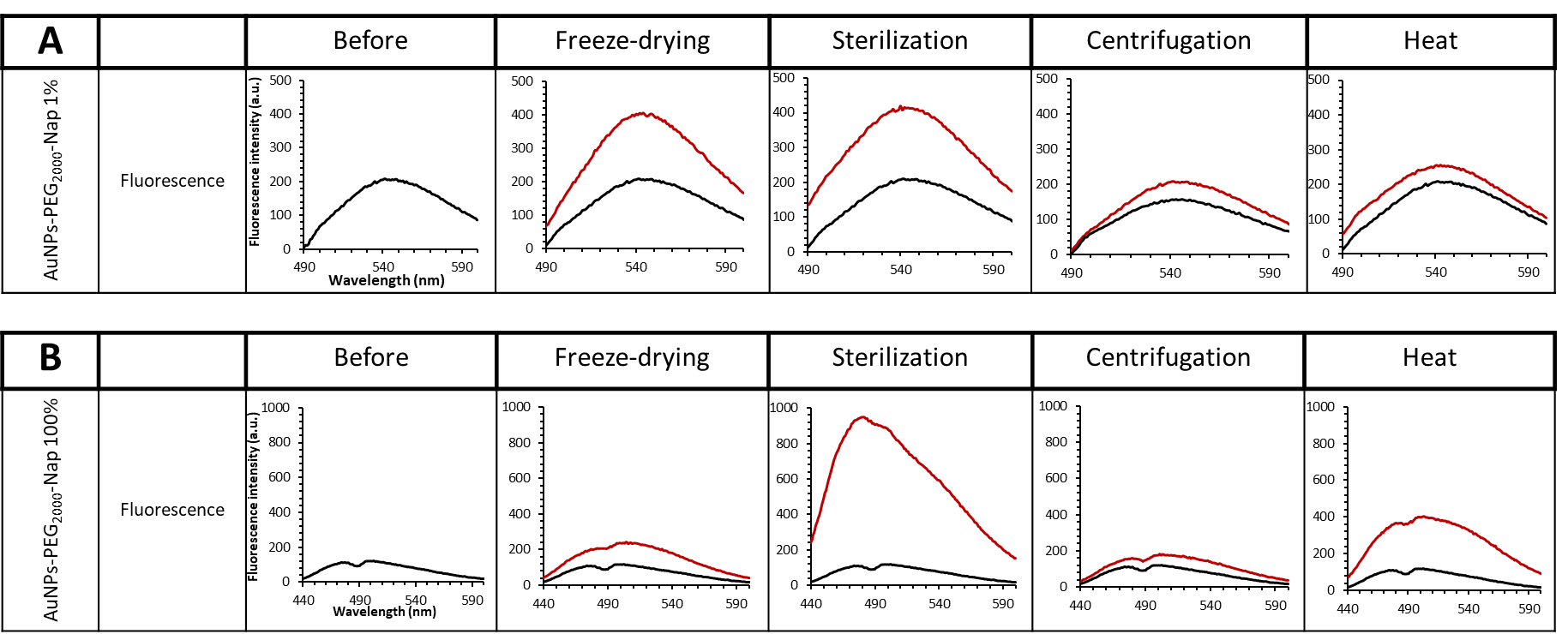


**Supplementary Figure 5.** Ultrastability characterization using fluorescence emission of (a) AuNPs-PEG_2000_-Nap 1% and (b) AuNPs-PEG_2000_-Nap 100% prior to treatment and after three cycles of 24-h freeze-drying, sterilization by autoclave, three precipitations by ultracentrifugation, and three periods of 12-h heating at 65°C. The fluorescence emission spectra of the AuNPs before and after the different treatments are represented by the solid black and solid red lines, respectively.
